# Electrochemical Fingerprints of Nanostructured Cu and Ag Electrodes

**DOI:** 10.1021/acselectrochem.5c00172

**Published:** 2025-07-15

**Authors:** Andrea Conte, Sara Bonacchi, Sabrina Antonello

**Affiliations:** Department of Chemical Sciences, 9308 University of Padova, Via F. Marzolo 1, 35131, Padova, Italy

**Keywords:** Nanostructured Cu electrodes, Ag electrodes, Pb UPD, OH^−^ electrosorption, cyclic voltammetry

## Abstract

Copper and silver electrodes, particularly in their crystalline and nanostructured forms, play a pivotal role in a wide range of electrochemical applications, including catalysis, sensing, and energy conversion. The electrochemical behavior of these materials, dictated by their crystallographic surface structures, has unique features and represents a key to understanding and optimizing their performance and expanding their applications. This review focuses on recent achievements about the electrochemical fingerprints of crystalline Cu and Ag electrodes, with particular attention to their nanostructured forms, and explores how surface-specific activities such as underpotential deposition (UPD), hydroxide electrosorption, and redox behavior vary across different surface orientations. The impact of crystal facet orientation on electrochemical performance is discussed, highlighting how variations in surface atomic configurations affect adsorption phenomena and, ultimately, catalytic selectivity. Understanding these structure–activity relationships is crucial for the design, synthesis, and characterization of innovative Cu and Ag nanostructures, with the aim of addressing the current challenges in renewable energy development.

## Graphical abstract

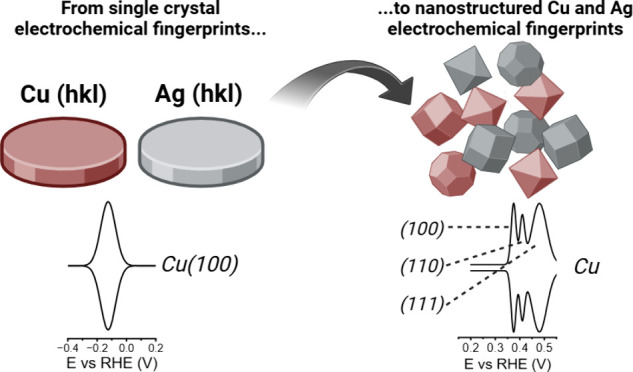

## Introduction

Climate change represents one of the most significant global challenges of the 21st century, driven primarily by the increase in greenhouse gases such as carbon dioxide (CO_2_) in the Earth’s atmosphere.^1^ This phenomenon is leading to the rise in global temperatures and increased frequency of extreme weather events.^2^ The urgency of mitigating climate change cannot be overstated, as its impact threatens ecosystems, human health, and the economy globally. In this context, the commitment of science has been directed, among other things, toward the exploration of molecules and materials that can act as efficient catalysts in the transformation of small molecules that play a key role in the energy production and pollution cycle, such as water splitting reactions, nitrate reduction reaction (NO_3_RR), and CO_2_ reduction reaction (CO_2_RR). Countless studies on homogeneous and heterogeneous catalysis have appeared in the scientific literature with the aim of finding a way to minimize the economic gap between the production of clean energy and the exploitation of fossil fuels or the optimization of production cycles aiming to minimize the generation of pollutants or greenhouse gases. Metal-based heterogeneous catalysis plays a pivotal role in addressing these environmental challenges, particularly in the context of industrial practices and sustainable development.^3,4^ Among metal catalysts, copper and silver are essential in the field of electrochemically activated catalysis.^5–18^ These metals possess unique chemical and physical properties that make them invaluable in various technological applications, including energy conversion,^19–22^ environmental remediation,^23,24^ sensors,^25^ and chemicals production.^26^

Cu is widely known for its excellent electrical and thermal conductivity, making it a critical material in wiring, electronics, and power transmission.^27^ In electrocatalysis, Cu versatility shines in the electrochemical reduction of CO_2_ and nitrates,^28,29^ where it facilitates the conversion of these molecules into valuable chemical products such as hydrocarbons,^30,31^ alcohols,^32^ and nitrogen-based compounds.^33–35^ Cu ability to adopt various oxidation states and form complex surface structures makes it a key candidate for catalyzing reactions that involve multielectron transfer processes.^36,37^ Ag is highly valued for its electrical conductivity, antimicrobial properties,^38^ and catalytic efficiency.^39^ In electrocatalysis, Ag plays a prominent role in reactions such as CO_2_RR, where it offers excellent selectivity for CO.^40–42^ Its relatively low cost compared to other noble metals and its efficiency in catalyzing reactions at low overpotentials make it an attractive material for fuel cells and CO_2_ reduction technologies.^43,44^ Together, Cu and Ag are crucial in advancing sustainable practices.^45,46^ Their distinct electrochemical properties allow for the fine-tuning of reaction pathways, enabling efficient, selective, and durable catalysts for next-generation energy and chemical processes.^45^

Over the past 50 years, fundamental research on single-crystal electrodes^47–50^ has paved the way for exploring more complex, industrially relevant high-surface-area materials.^20,51^ Monometallic low-index (*hkl*) single crystals, such as the face-centered cubic (fcc) metal surfaces (111), (110), and (100), offer atomically flat, well-ordered surfaces with highly periodic surface atomic structures.^52^ These crystals enable the precise investigation of specific surface sites in a controlled environment. On the other hand, nanocrystal structures have garnered significant attention in electrochemistry due to their unique catalytic properties, high surface areas,^53^ and tunable morphologies.^54,55^ Nanostructures based on Cu have been already widely explored for their applications in various electrochemical processes, including NO_3_RR,^34,56–58^ hydrogen evolution reaction (HER),^59,60^ and CO_2_RR.^7,61^ However, the complexities of nanostructured materials compared to bulk Cu require more refined and concerted approaches to thoroughly understand their mechanism of action. Surface analysis techniques such as ultrahigh-vacuum (UHV) scanning probe microscopy and XANES are often technically and economically demanding and in many cases examine only a very limited surface area and localized features. While these techniques provide valuable and irreplaceable information, this review emphasizes the usefulness of an older, inexpensive, yet still highly effective experimental approach such as cyclic voltammetry (CV). CV provides an accessible tool for obtaining affordable fingerprints of metal surface structures, and this review, which compiles information about the influence of chemical and electrochemical surface treatments on exposed crystal patterns and defectiveness, aims to contribute to a deeper understanding of how to tailor Cu- and Ag-based electrodes for different electrochemical applications.

This survey conveys to the reader that electrochemical surface investigation techniques, such as underpotential deposition (UPD) and hydroxide electrosorption, can drive exciting advancements, particularly in emerging fields such as nanostructured electrocatalysts with precisely exposed crystal facets.

## Electrochemical Characterization of Crystalline Cu Electrodes and Cu-Based Nanomaterials

Over the past few years, the electrochemical characterization of crystalline Cu electrodes has gained renewed significant importance driven by the potential use of this metal as catalyst for the electroreduction of nitrate,^28,34,56,57^ CO_2_,^62–67^ and CO.^68^ The efficiency and selectivity of such reactions have shown an impressive sensitivity to the crystalline structure of the exposed surface,^69^ and therefore, correlating reactivity, selectivity, and crystalline pattern has become of fundamental relevance and interest. In this framework, CV represents a convenient approach to gather a series of essential properties of the electrocatalytic surface such as (i) the electrochemically active surface area (ECSA), (ii) the exposed facets and crystallographic structure, and (iii) the presence and structure of surface defects.

It is indeed well-established that Cu electrodes with different crystallographic orientations exhibit, in alkaline solutions, distinct redox behaviors within a potential range delimited by hydrogen evolution and the onset of irreversible Cu oxidation with the formation of Cu_2_O and CuO/Cu­(OH)_2_. This range usually spans between −0.25 and 0.5 V vs RHE and is related mostly to the adsorption of OH^–^ on the different Cu crystal facets.^47,70^ The oxidation/reduction rate, including electrode restructuring and corrosion,^71^ and the electrocatalytic properties are similarly affected.^72,73^

The interaction of OH^–^ with Cu is highly sensitive to the crystal facet’s structure, the presence of large terraces, and defects or steps. In voltammetry, the formers are revealed as signals in a potential range between −0.2 V and +0.2 V vs RHE. Moreover, the peaks related to OH^–^ adsorption/desorption, observed in the region between +0.32 V and +0.50 V vs RHE, have been attributed to the oxidation of OH^–^ to adsorbed oxygen species, which is very evident in the case of Cu nanocrystals due to the presence of defects on the surface. This sensitivity is crucial, as it provides a bulk average perspective of the material’s surface, which is rare in surface analysis techniques. Figure 1 shows a cartoon of what happens at the Cu–solution interface when the electrode is polarized at two different potentials and the processes of adsorption and desorption of OH^–^ take place.

**Figure 1:**
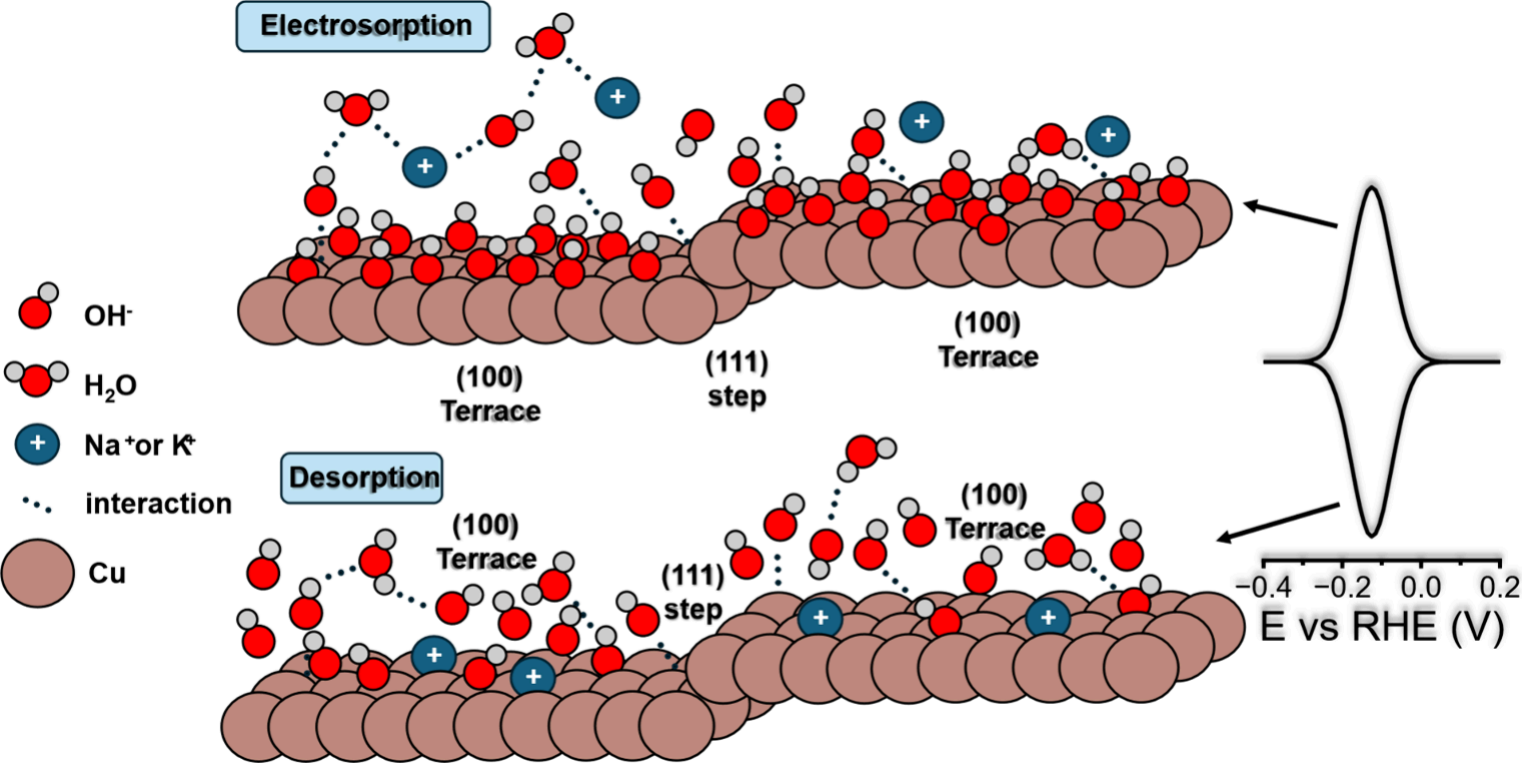
Illustration of the Cu(100) electrode during the electrosorption of OH^–^ in alkaline media.

As said, different crystal facets of Cu (e.g., Cu(111), Cu(100), Cu(110)) show distinct CV features due to their different surface energies (Figure 2)^70^ which affect their interaction with hydroxide ions. The peak potential reflects the reactivity order and the binding energy of OH^–^, with Cu(110) > Cu(100) > Cu(111), which in turn scales with the characteristic work function of the metal surface.^70^ The Cu(111) face is generally the most stable and shows the least reactivity.

**Figure 2:**
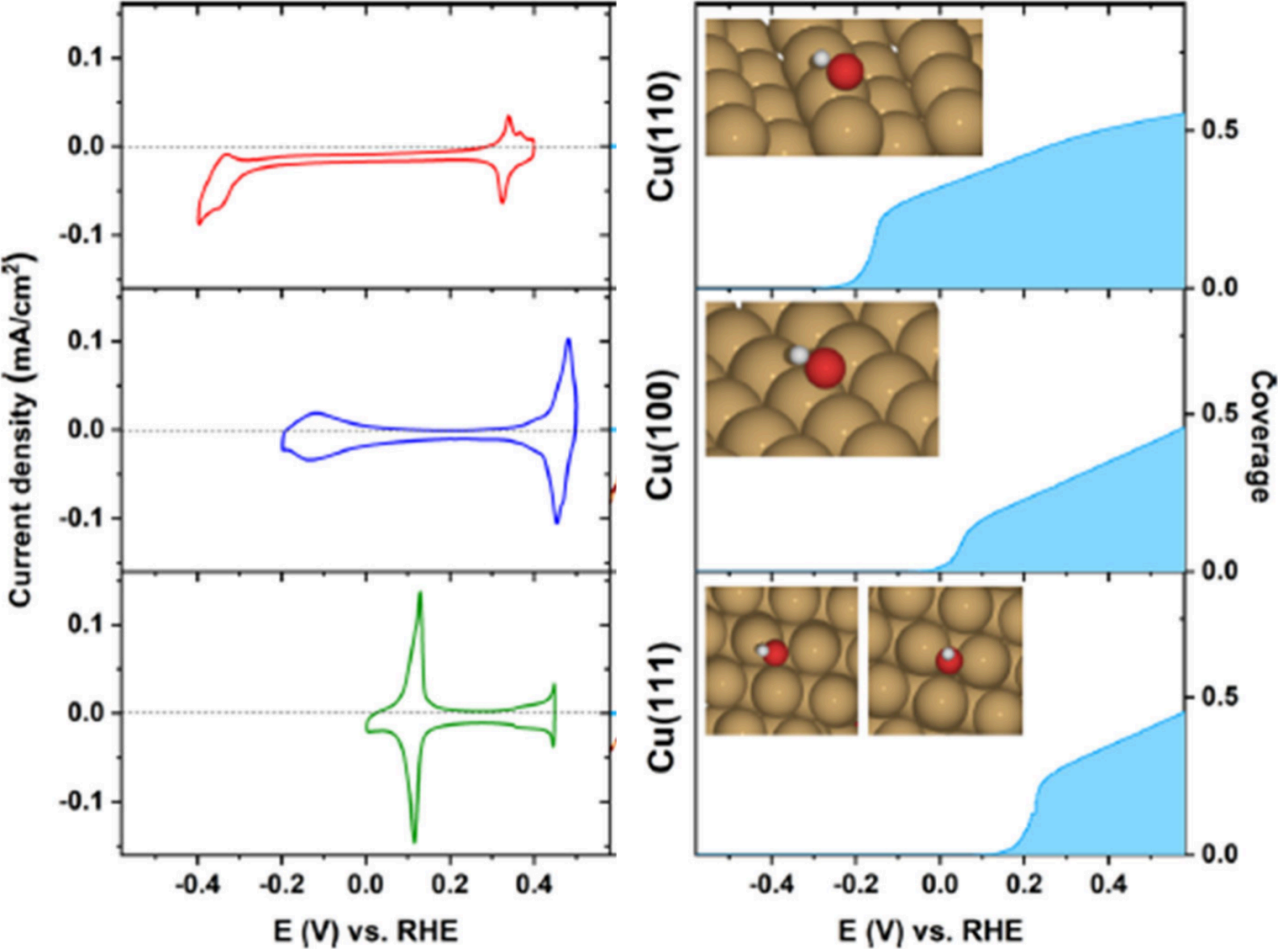
Experimental CVs (left) for Cu(110), Cu(100), and Cu(111) illustrate facet-dependent OH adsorption/desorption features in Ar-saturated 0.1 M KOH electrolyte at a scan rate of 50 mV/s. Calculated OH coverages (right) as a function of applied potential. The insets illustrate the optimal adsorption geometries of OH^–^ under these conditions. Reproduced from ref 70. Copyright 2020 American Chemical Society.

The magnitude of the peaks, particularly their integral, is related to the number of electrons exchanged in the process, which in turn depends on the surface coverage of adsorbed hydroxide. Typical coverage values range from 0.1 to 0.5 monolayers depending on the conditions. All the aforementioned peak–crystal facet assignments were accomplished by investigation of single-crystal electrode materials,^70^ which represents a fundamental starting point to analyze the plethora of differently shaped nanomaterials that synthetic advancements continue to produce. Only when the Cu electrode is a single crystal prepared under very controlled conditions^47^ are well-defined peaks observed and a certain attribution can be performed. With this information, both polycrystalline bulk materials and nanomaterials can be investigated. In the first case, particular attention has to be paid to the surface preparation and cleaning method since they have a relevant effect on the voltammetric pattern.

Before discussing the different outcomes on surface treatments on Cu polycrystalline and single-crystal electrodes, two very classical works on characterization of Cu single-crystal surfaces from Hori’s group are noteworthy to mention, being very inspiring and full of interesting information from our point of view.

In the first one, Koga et al. investigated the charge displacement associated with CO adsorption on copper single-crystal electrodes from the [110] zone, with a particular focus on the relationship between surface structure, adsorption behavior, and electrochemical response.^74^ Using cyclic voltammetry in phosphate buffer solution (pH 6.8) at 0 °C, the authors observed characteristic redox waves on Cu surfaces exposed to CO, which were absent on the (111) facet. These redox features result from a reversible charge displacement process where specifically adsorbed phosphate anions (H_2_PO_4_^–^) are displaced upon CO adsorption. The shape, position, and intensity of these redox waves depend strongly on the crystallographic orientation of the surface and serve as electrochemical fingerprints of different Cu facets. A clear linear correlation was found between the potential of the redox peak and the work function of the surface, suggesting a strong link between the electronic properties of the metal and CO adsorption. Furthermore, the magnitude of the charge transfer is correlated with the density of (100) sites, implying that anion adsorption preferentially occurs at these sites, with each anion occupying three to four Cu atoms. This work highlights the utility of CO voltammetry as a diagnostic tool for probing surface structure on Cu electrodes, drawing analogies to the role of hydrogen adsorption on Pt, and contributes to a deeper understanding of surface–adsorbate interactions relevant to electrocatalysis and CO_2_ reduction.

The second work by Koga et al. explored the electrochemical adsorption behavior of CO on low-index copper surfaces (Cu(100), Cu(111), and Cu(110)) in acidic media using voltammetry and charge displacement analysis.^75^ The study revealed that on Cu(100), a distinct redox peak appeared in the CV in the presence of CO, which is absent on Cu(111) and Cu(110), indicating structure-sensitive CO adsorption. This redox feature was attributed to a reversible, net charge-neutral adsorption process involving the displacement of specifically adsorbed anions (e.g., sulfate or phosphate) by CO. The authors demonstrated that the charge and position of this redox peak was consistent with a model where CO adsorption replaces anions without electron transfer to the CO molecule itself.^75^ Furthermore, comparisons across the three surfaces highlight that CO preferentially adsorbs on Cu(100), correlating with its higher density of fourfold hollow sites. This behavior mirrored previous observations in neutral solutions and reinforced the role of surface crystallography in dictating the adsorption behavior. Overall, the study emphasized the use of CO as a sensitive probe for characterizing Cu surface structures via voltammetric signatures, with implications for understanding structure–reactivity relationships in electrocatalysis.^75^

More recently, Engstfeld et al.^76^ investigated the influence of various pretreatments on the crystallographic orientation of Cu electrodes by conducting experiments on both polycrystalline Cu and Cu(100) single-crystal electrodes. The diverse peak patterns observed in KOH suggest that the crystallographic orientation of each sample was unique. Even two foils from the same supplier with identical specifications can exhibit distinct voltammetric features, as demonstrated in Figure 3A and Figure 3E, both of which were electropolished. Further, pretreating Cu foil by radiofrequency heating in a hydrogen atmosphere alters its crystallographic orientation compared to the same electropolished sample. Sequential pretreatments involving electropolishing followed by radiofrequency heating or vice versa also result in changes in crystallographic orientation relative to the solely electropolished electrode. Interestingly, the radiofrequency-heated and then electropolished sample (Figure 3C) exhibits features that are more pronounced than those of the original radiofrequency-heated sample, suggesting that the electropolishing process is sensitive to the initial crystallographic orientation of the electrode surface. Finally, a mechanically polished Cu­(poly) electrode is indistinguishable from a mechanically polished Cu(100) single crystal (Figure 3G,H). This similarity can be attributed to the comparable surface structures obtained by identical polishing.

**Figure 3:**
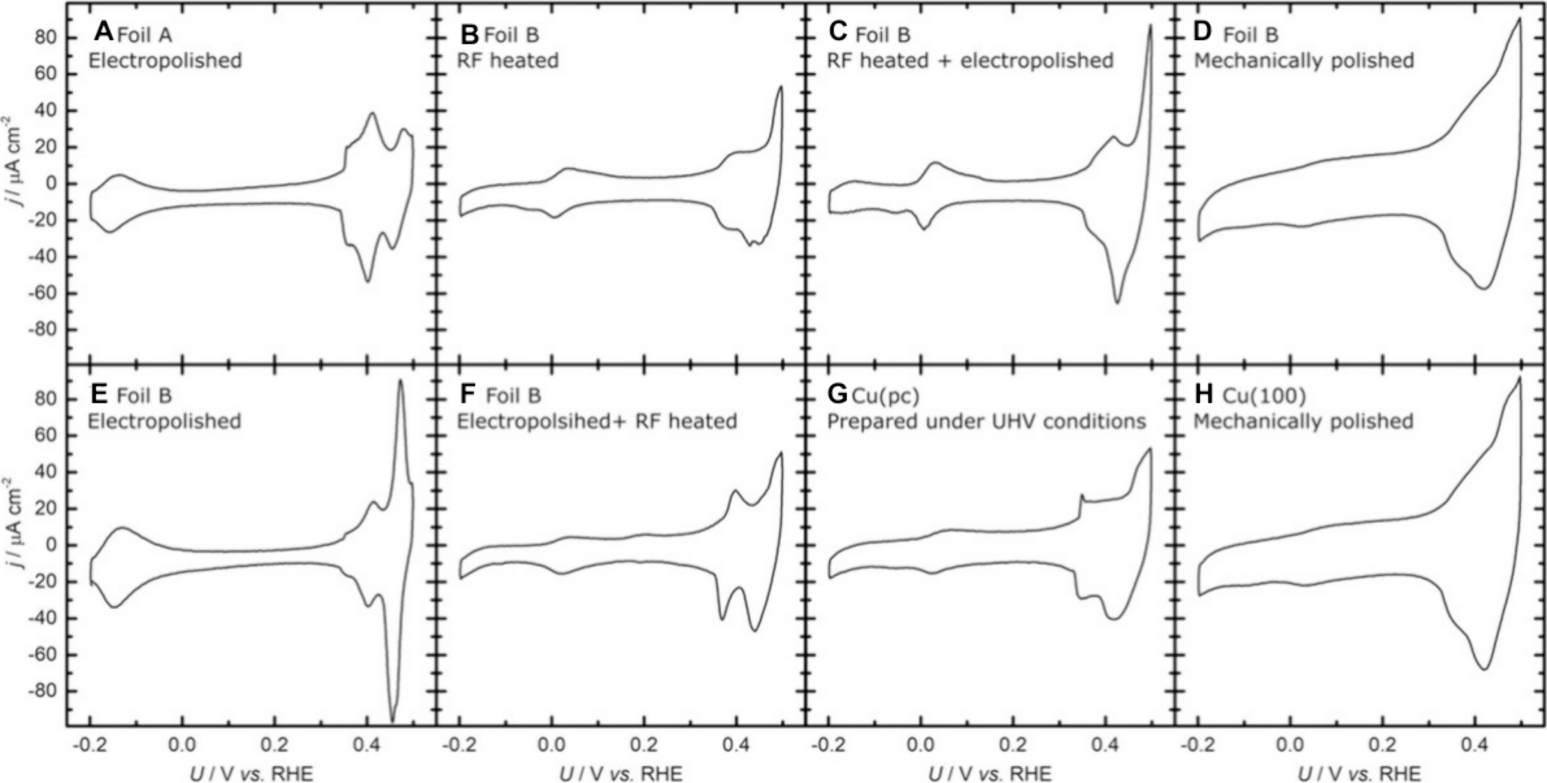
CVs recorded on various Cu electrodes exposed to different pretreatments in 0.1 M KOH at a scan rate of 50 mV s^–1^. The CVs are referred to as (A) electropolished Cu foil A, (B) electropolished Cu foil B, (C) radiofrequency-heated Cu foil B, (D) electropolished and subsequently radiofrequency-heated Cu foil B, (E) radiofrequency-heated and subsequently electropolished Cu foil B, (F) Cu­(poly) sample prepared under UHV conditions, (G) mechanically polished Cu foil B, and (H) mechanically polished Cu(100). Reproduced with permission from ref 76. Copyright 2018 Wiley-VCH.

More recently, Koper’s group proposed an induction annealing technique for preparing Cu single crystals and polycrystalline surfaces to achieve clean, reproducible voltammetry with distinct facet-specific features.^77^ This method, consisting of rapid heating of the sample in an inert or reducing atmosphere followed by controlled cooling, was leveraged to address inconsistencies in surface preparation observed with conventional electropolishing. The approach produced surfaces with well-defined adsorption behaviors on Cu(111), Cu(100), Cu(110), and polycrystalline Cu. Compared to electropolishing, induction annealing minimizes surface defects, improves reproducibility, and stabilizes the surface over multiple experimental cycles, though it falls slightly short of the quality achieved using UHV techniques. Facet-specific OH^–^ adsorption at +0.1 V for Cu(111), −0.125 V for Cu(100), and +0.33 V for Cu(110) was clearly identified. This method also ensures a consistent facet distribution on polycrystalline surfaces, enabling reliable studies of overall catalytic properties. Importantly, the induction annealing approach bridges the accessibility gap between UHV methods and traditional electropolishing, offering high-quality surfaces without the need for expensive setups. This reproducibility and scalability are vital for advancing studies in CO_2_ electroreduction, where facet-specific reactivity plays a key role in determining product selectivity, such as ethylene formation being more favorable on Cu(100) at lower overpotentials compared to Cu(111) and Cu(110). Despite the improved surface preparation, challenges remain in achieving defect-to-terrace ratios comparable to those of UHV-prepared surfaces and in understanding the influence of electrolytes on voltammetric behavior. In previous investigations, Koper’s group also identified the electrochemical fingerprints of higher-index crystal planes in alkaline solutions.^47^ Their sound analysis highlighted that Cu(911) shares similar electrochemical features with Cu(100), which has a similar structure but shows lower peak intensities correlated to the lower dimension of the corresponding terraces, whereas Cu(322) shows peaks similar to those of Cu(111).

The Cu single-crystal electrodes were also investigated according to 1990 literature through scanning tunneling microscopy (STM), which provided essential information about atomic disposition and organization of the surface. A noteworthy work by Kunze et al. presented a detailed study of the atomic-scale surface structure and dynamics of Cu(111) in 0.1 M HCl using in situ STM.^78^ They showed that under electrochemical conditions, the Cu(111) surface exhibited well-ordered terrace–step–kink structures with monoatomic steps and that step orientations were not random but preferred energetically favored directions such as ⟨110⟩. The study also observed potential-dependent changes in surface morphology: at more negative potentials, the surface remained stable and well-ordered, while at more positive potentials, enhanced mobility of surface atoms led to dynamic changes, including step fluctuations and increased kink density. These results demonstrated that the electrochemical environment can significantly influence surface structure and dynamics at the atomic level, offering insights into how step orientation and reactivity evolve under real operating conditions.

Wilms et al. explored the influence of chloride adsorption on the atomic-scale structure and dynamics of Cu(111) surfaces using in situ STM in 10 mM HCl.^79^ They observed that adsorbed Cl^–^ forms a well-ordered (√3×√3)­R30° structure, which induces a strong restructuring of the surface: initially random step orientations rearrange to favor the {211} direction, corresponding to the close-packed rows of Cl atoms. This reordering occurs rapidly (within 30 min), indicating high surface mobility of Cu atoms. The study also revealed dynamic processes such as step bunching and terrace reorganization, driven predominantly by surface diffusion rather than diffusion through the electrolyte. Notably, Cl adsorption significantly enhanced surface atom mobility and reduced step edge barriers, as evidenced by the decay and redistribution of a multilayer Cu pile over time. The work highlighted how adsorption of a specific anion (in this case Cl^–^) not only alters step orientation but also facilitates mass transport at the surface, providing insight into the atomistic mechanisms that govern electrode surface evolution under electrochemical conditions.

In 2006, Broekmann et al. investigated the structure and dynamics of Cu(111) surfaces in the presence of chloride and iodide ions under electrochemical conditions using a combination of in situ STM and surface X-ray diffraction (SXRD).^80^ The study revealed that both Cl^–^ and I^–^ induce distinct surface reconstructions: Cl^–^ promotes the formation of a well-ordered (√3×√3 )­R30° adlayer, while I^–^ leads to an I/Cu(100)-c(2×5) reconstruction. These adsorbates not only restructure the surface at the atomic level but also significantly influence step edge morphology and dynamics, with iodide causing greater step roughening and mobility. The work highlights the interplay between halide adsorption and surface atomic structure, providing insight into the mechanisms by which adsorbates modulate the catalytic properties and reactivity of copper electrodes in electrochemical environments.

After these pioneering works on Cu electrochemical fingerprints, an analogous characterization approach started to be applied to electrodes based on nanostructured Cu samples to identify their predominant surface pattern or estimate the relative amount of surface defects. Zhang et al. analyzed various Cu oxide catalysts characterized by a nanosheet geometry.^81^ As shown in Figure 4A, CV measurement on pristine CuO reduced nanosheets (o-Cu) showed a prominent peak at approximately +0.44 vs RHE, corresponding to the OH^–^ adsorption on Cu(111), whereas the CV of plasma-treated reduced Cu nanosheets (p-Cu) displayed a peak at approximately +0.33 V, in agreement with OH^–^ adsorption on Cu(100) facets. Quantification of the percentages of the two facets via integration of the Cu­(OH)_ad_ peaks yielded 96.2% Cu(111) on o-Cu and 95.7% Cu(100) on p-Cu (Figure 4B,C). The quantitative analysis further showed that the formation of Cu(110) facets during the CO_2_RR is very limited, merely 2.0% for o-Cu and 1.1% for p-Cu. In addition, X-ray diffraction patterns showed that the strongest diffractions for o-Cu and p-Cu were at 43.3° and 50.4°, indicating the preferential exposure of (111) and (100) facets, respectively. These results, together with structural characterizations above, confirm that o-Cu is dominated by (111) while p-Cu mainly exposes (100).

**Figure 4:**
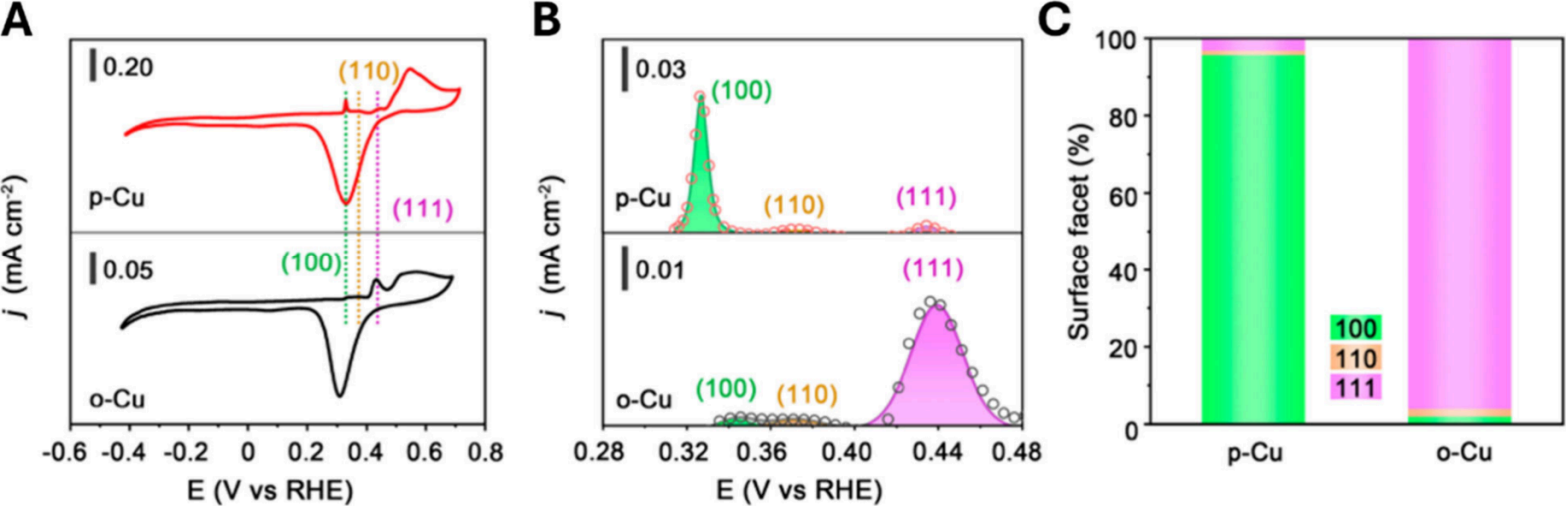
(A) CV curves of o-Cu and p-Cu in 1.0 M KOH. Scan rate: 20 mV s^–1^. (B) Fitting and integration of OH^–^ adsorption peaks of o-Cu and p-Cu. (C) Ratio of Cu(100), Cu(110), and Cu(111) facets quantified by OH^–^ electrosorption. Reproduced from ref 81. CC BY-NC-ND 4.0.

A recent study by Chen et al.^82^ highlights a novel approach to fabricate Cu-based electrocatalysts through the electrochemical activation of three-dimensional (3D) flowerlike CuO micro/nanostructures grown in situ on Cu foils and subsequently modified with surfactants. This method yields remarkable control over the surface properties and crystal facet exposure, as shown in the CV analysis in Figure 5A, where four samples are compared: the pristine Cu foil, the sample obtained by reduction in NaBH_4_ (Cu-F), the flowerlike Cu modified with hydrophobic treatment (Cu-F-M), and the one annealed in air (Cu-P-M). The CV curves reveal distinct OH^–^ adsorption potentials for different exposed Cu facets, specifically (100), (110), and (111). Importantly, CV peak deconvolution revealed an exceptionally high exposure ratio (65%) for Cu(100) facets for Cu-F-M that is significantly higher than those observed in other Cu-based samples. The exposure ratio of Cu(100) facets follows then the descending order Cu-F-M > Cu > Cu-P-M > Cu-F. This remarkable facet orientation is further distinguished by the nearly complete disappearance of Cu(111) planes in the Cu-F-M sample after surface hydrophobic modification. The preferential exposure of Cu(100) facets in Cu-F-M was attributed to the interplay between the surface energy dynamics and surfactant interactions during electrochemical activation. The Cu(100) facet, with its lower surface energy compared with other crystal planes, likely serves as a preferred nucleation site for the carboxylate ions of sodium laurate. These ions selectively coordinate with Cu(100) atoms, thus effectively stabilizing this facet. Simultaneously, the exposed alkane chains of the laurate ions on the Cu­(100) plane impede the growth of competing Cu(110) and Cu(111) facets. This effect contrasts sharply with the behavior of the Cu-F sample, which does not undergo such targeted facet orientation modification. As a result, the Cu-F-M sample exhibits a significant shift in the crystal plane orientation, culminating in the dominant exposure of Cu(100) facets.

**Figure 5:**
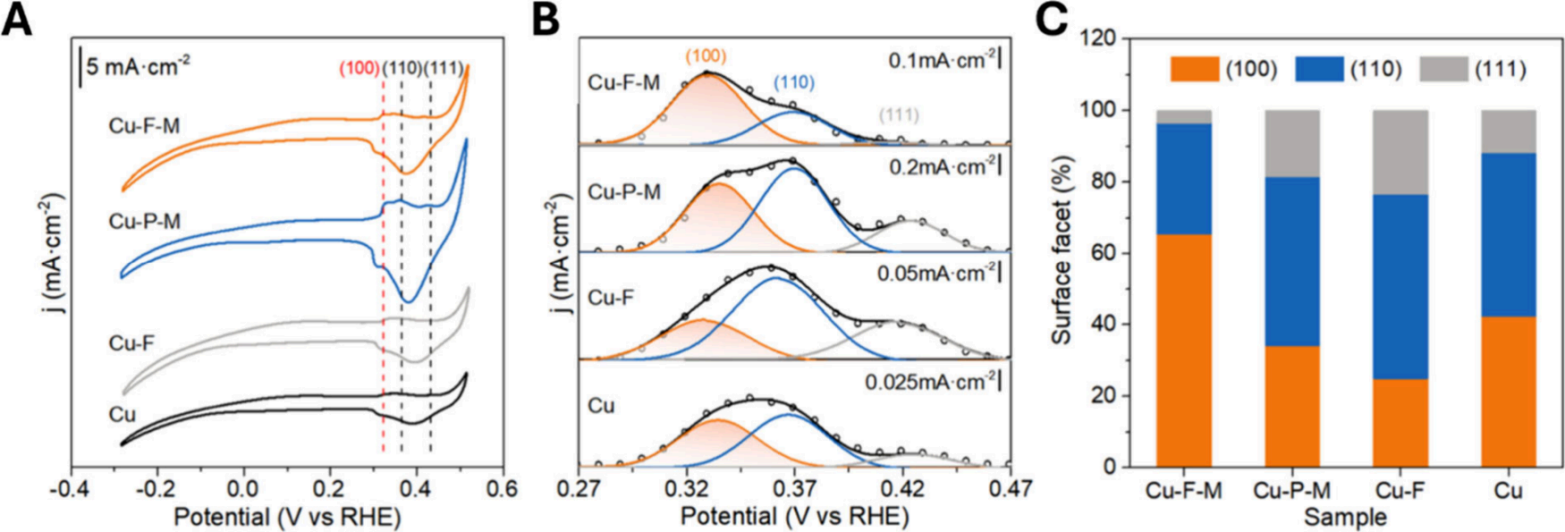
(A) CV curves of the Cu samples in 1.0 M KOH with a sweep rate of 100 mV s^–1^. (B) Deconvolution of OH^–^ adsorption peaks referred to the different Cu facets of the samples. (C) Relative amounts of exposed surface Cu facets of the samples quantified by OH^–^ adsorption. Reproduced from ref 82. Copyright 2024 American Chemical Society.

Xie et al. developed a Cu nanocubes catalyst that upon chemical treatment with NaBH_4_ and/or electroreduction exhibited specific crystal planes on the surface (Figure 6A).^83^ The cubes obtained by electroreduction (OD-Cu) and by using different quantities of NaBH_4_ (PROD-Cu-I, -II, -III, -IV) (Figure 6B) were analyzed by CV in Ar-saturated 1 M KOH to evaluate the surface facet distribution. The OH^–^ adsorption peaks at approximately +0.35, +0.38, and +0.45 V vs RHE appearing in the CV curves of PROD-Cu-I, -II, -III, and -IV represented the low-index facets of Cu(100), Cu(110), and Cu(111), respectively. Compared with OD-Cu, which only shows the (100) peak, the additional signals revealed the presence of facets with higher energy on PROD-Cu-I, -II, -III, and -IV surfaces. There was no evidence of Cu(111) in the OD-Cu, while it appeared in PROD-Cu-I (15.5%). The percentage of Cu(110) (32.7%) increased with the decrease of Cu(100) (51.8%), meaning that the generation of Cu(111) facilitates the formation of Cu(110) in this situation. This phenomenon further proceeds as the amount of NaBH_4_ is increased, with the percentage of Cu(110) showing a fluctuation from 41.4% (PROD-Cu-II), to 41.5% (PROD-Cu-III), and to 38.9% (PROD-Cu-IV). The increasing amounts of Cu(111) facets in PROD-Cu-I, -II, -III, and -IV was attributed to its low surface energy and high stability that hampers the formation of other facets.

**Figure 6:**
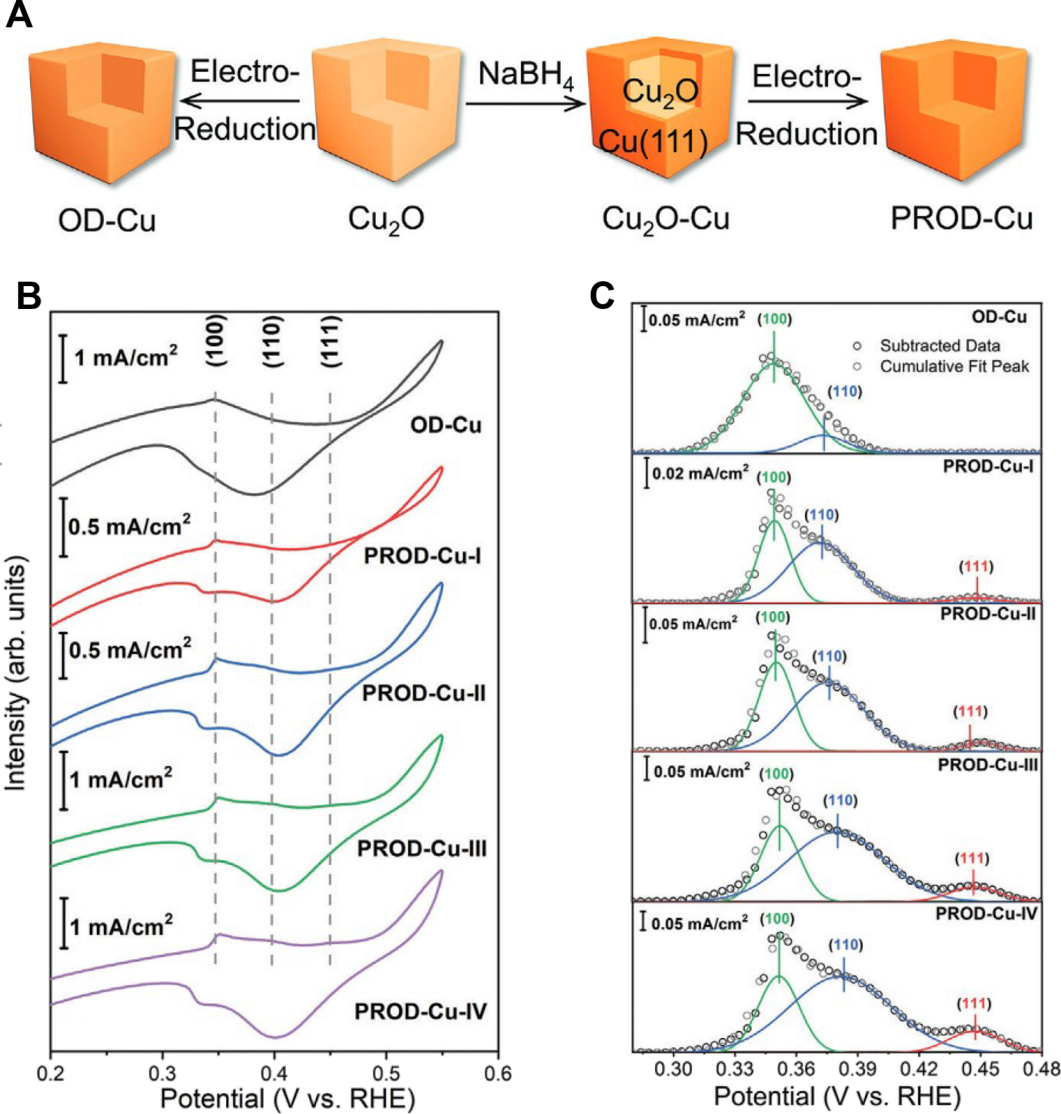
(A) Schematic representation of different surface treatments of Cu_2_O nanocubes. (B) CV results in 1.0 M KOH. (C) Deconvolution of OH^–^ adsorption peaks. Reproduced with permission from ref 83. Copyright 2024 Wiley-VCH.

Cao et al. reported in 2017 a systematic investigation of CO_2_ reduction on highly dense Cu nanowires (CuNWs), with the focus on understanding the surface structure effects on the formation of absorbed CO and the evolution of gas-phase CO product at low overpotentials (more positive than −0.5 V).^44^ Figure 7A summarizes the CVs recorded for the five types of Cu nanowires, where ECR refers to electrochemically reduced CuNWs and HR-150, HR-200, HR-300 (1 h), and HR-300 (15 h) refer to Cu mesh samples reduced by annealing the oxidized Cu at 150 °C for 15 h, at 200 °C for 1 h, at 300 °C for 1 h, and at 300 °C for 15 h in a flow of forming gas (5% H_2_/Ar, 20 sccm), respectively. A series of peaks appear in the anodic scans prior to the onset of irreversible oxidation at approximately +0.5 V. The peaks at ca. +0.36, +0.4 to +0.42, and +0.44 to +0.46 V can be assigned to the OH^–^ features associated with the (100), (110), and (111) facets of fcc Cu, respectively. The slight shifts in peak positions among the different types of CuNWs are likely a result of the varying surface roughness factors or the capacitance effect. In addition to these three peaks, two other peaks were observed at ∼0.1–0.15 and ∼0.32–0.34 V, which were not reported in previous studies on extended surfaces. In particular, the peak at ∼0.32–0.34 V (denoted with * in Figure 7A) was observed to become more pronounced for the CuNWs prepared at higher temperatures. To investigate potential assignments for these two peaks, DFT calculations were performed for the binding energies of OH on various Cu facets. The calculations showed that (110) and (100) bind OH more strongly than (111) by 14.3 and 16.3 kJ/mol, respectively, which is consistent with the above observation of OH^–^ peaks at lower potentials (Figure 7B). Facets with higher indexes typically bind to OH^–^ even more strongly than these low-index facets, e.g., (211) has a binding energy ∼38 kJ/mol higher than that of (111). The low-coordination step sites on these facets are likely to be more oxophilic than the terrace sites and give rise to the low-potential peak at ∼0.1–0.15 V. They had previously assigned the * peak at ∼0.32–0.34 V to the (211) or (110) reconstructed facets (Figure 7A).

**Figure 7:**
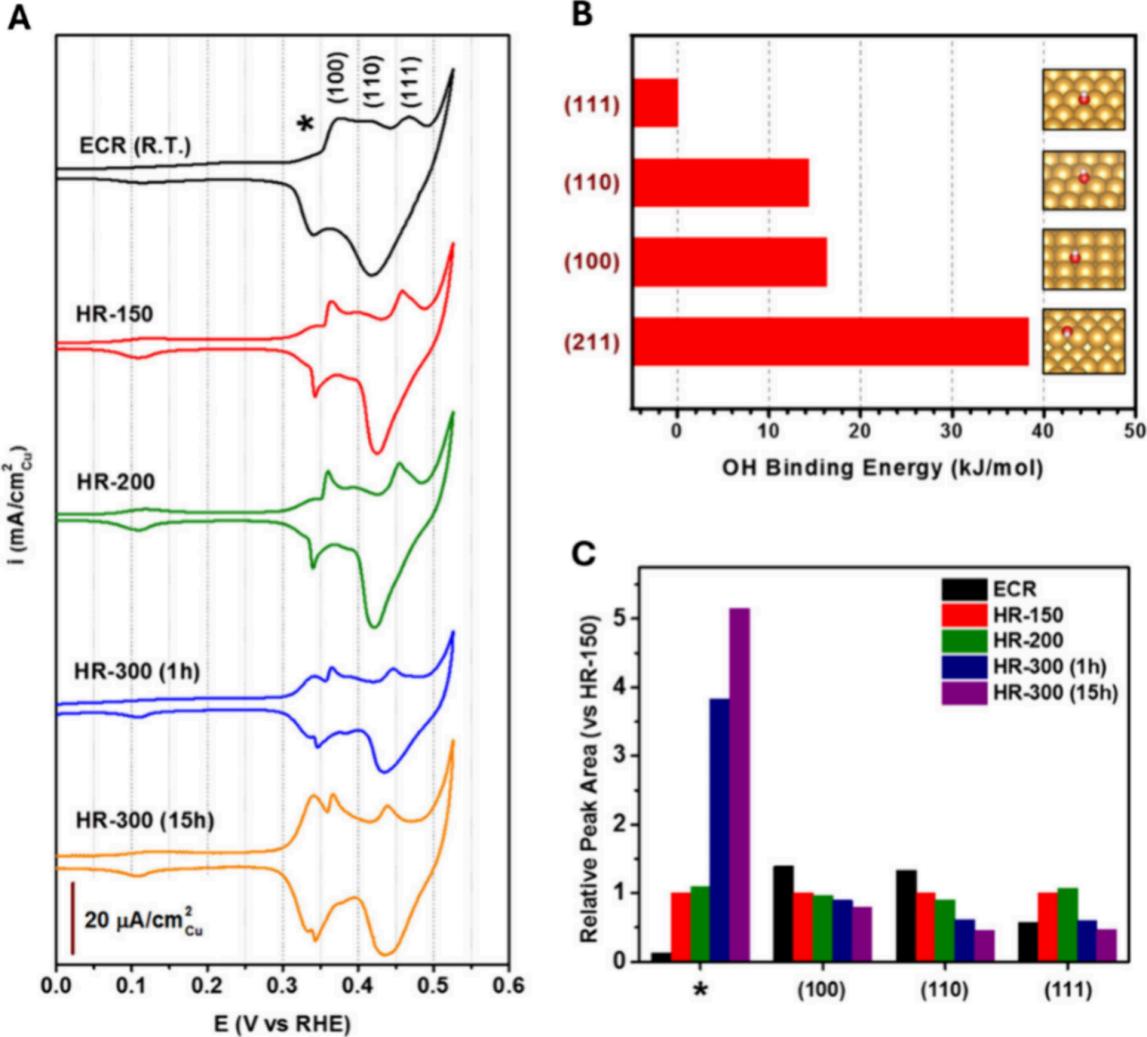
Surface structure analysis for the CuNWs. (A) CVs recorded in 1.0 M KOH, capturing the surface-specific OH^–^ adsorption. (B) Calculated OH binding energies for the Cu facets referred to as Cu(111). (C) Relative peak areas derived from the deconvolution for the CV peaks. Reproduced from ref 44. Copyright 2017 American Chemical Society.

Very recently, our group explored the systematic design and synthesis of CuNWs with controlled morphological features to enhance their performance as transparent electrodes and electrocatalysts for CO_2_ reduction.^73^ CuNWs are recognized for their excellent conductivity and potential for customization in specific electronic applications. The study aimed to master the synthesis and morphological features of CuNWs using a Design of Experiments (DOE) approach and explore different synthetic parameters of a hydrothermal method (Cu source, temperature, reaction time, surfactant type, and concentration) to optimize control over the CuNWs’ structure and properties. A multivariate DOE approach was used to minimize experimental efforts and generate predictive models correlating the CuNWs’ morphology with their electrochemical behavior. We demonstrated that CuNWs highly rich in (100) and (110) surface defects, detected by CV, drive the selectivity of CO_2_ electroreduction toward ethylene. Recently, in addition to pure Cu catalysts, alloys and bimetallic systems have been increasingly utilized in the CO_2_RR. Notably, a study by Li et al. demonstrated a method to boost ethanol production in the CO_2_RR by modifying surface oxophilicity through a Cu–Sn bimetallic system. Surface oxophilicity is crucial in reactions involving oxygen-containing intermediates, as it affects the binding strength of these species on catalysts.^84^ The authors initially focused on understanding how oxophilicity changes with different CuSn_*x*_ catalyst compositions using CV tests in 0.1 M NaOH solution. The binding strength of adsorbed OH^–^ is indeed a direct indicator of oxophilicity. The CV analysis revealed distinct behavior between the bare Cu and CuSn_*x*_ catalysts. Bare Cu showed typical redox peaks of Cu^0^–Cu^+^ at 0.328 and 0.577 V vs RHE (Figure 8B) and a peak for OH^–^ adsorption at 0.360 V vs RHE. This signals on bare Cu were similar to those seen on polycrystalline Cu, with a prominent peak corresponding to the Cu(100) facet followed by less pronounced peaks for Cu(110) and Cu(111). Introduction of Sn significantly altered this pattern: the CuSn_*x*_ catalysts displayed a single, more intense peak that shifts to lower potentials, suggesting stronger OH^–^ adsorption and increased oxophilicity. The maximum adsorption strength was observed on the CuSn_0.025_ catalyst, where the peak onset and vertex moved to 0.343 and 0.352 V vs RHE, respectively. Increasing the Sn content further resulted in a decrease in the peak intensity and the emergence of a broad peak around 0.150 V vs RHE, associated with Sn’s stronger affinity for oxygen. For catalysts with higher Sn concentrations, such as CuSn_0.25_, only a broad peak between 0.100–0.200 V vs RHE was evident, indicating predominant OH^–^ adsorption on Sn sites. These findings underscore that Sn not only enhances the oxophilicity of Cu sites but also introduces new, more oxophilic Sn sites. A clear relationship between the oxophilicity of Cu sites and ethanol production was observed.

**Figure 8:**
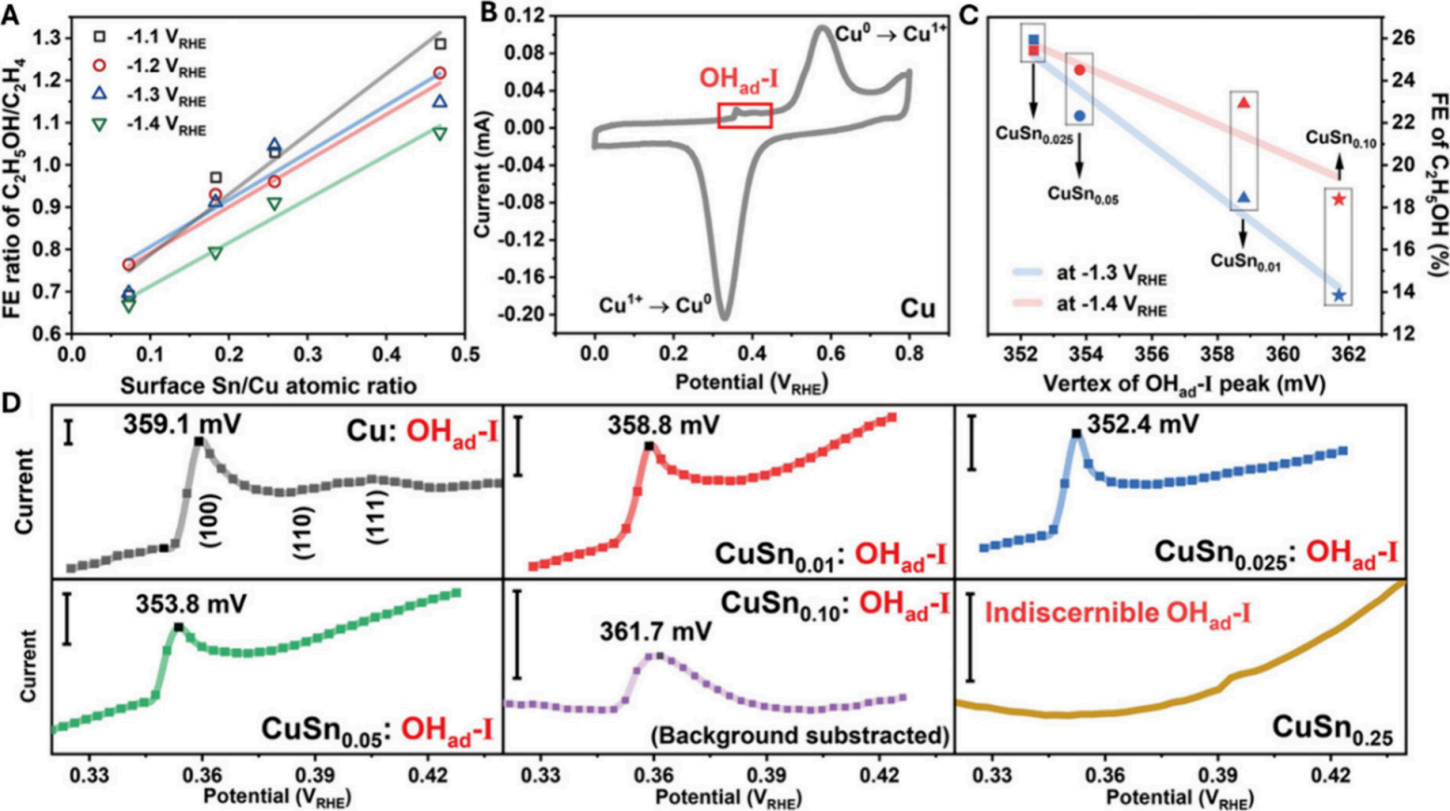
Selectivity correlation and surface oxophilicity characterization. (A) Correlation between the surface Sn/Cu atomic ratio and the FE ratio of C_2_H_5_OH/C_2_H_4_. (B) CV curves of bare Cu catalyst in 0.1 M NaOH, at 10 mV s^–1^. (C) Linear correlations of the oxophilicity of Cu sites with FE of C_2_H_5_OH at −1.3 and −1.4 V RHE. (D) Enlarged CV curves showing OH^–^ adsorption peaks for Cu and CuSn_*x*_ catalysts. Reproduced from ref 84. CC BY 4.0.

It is noteworthy to mention a recent work by Bagger et al. dealing with both theoretical and experimental analysis and offering a comprehensive study aimed at understanding the atomistic interactions between Cu surfaces and various electrolytic environments.^85^ By combining ab initio molecular dynamics (AIMD) simulations with experimental validations, the study explored how adsorbed species such as H*, OH*, and Cl* affect the electrochemical behavior of Cu across different crystal facets under acidic (HClO_4_), neutral (KHCO_3_), and alkaline (NaOH) conditions. The results from the simulations align closely with experimental CVs, providing a robust validation of the computational approach. This alignment reveals crucial insights into the binding energies and electrochemical responses of Cu, which are significantly impacted by the nature of the electrolytic environment. This integration of theoretical predictions with empirical data underscores the potential of computational models in the development of advanced catalytic systems.

Choi et al.^53^ reported a fascinating example involving the modification, through electrochemical reduction at −1.05 V vs RHE, of CuNWs for enhanced electrochemical performance. After the treatment, characteristic CV peaks that were assigned to high-index facets were observed at about +0.30 V vs RHE when 0.1 M KOH was used as the electrolyte. High-index facets are known for their increased atomic step density, which can provide more active sites for catalytic reactions.^86^ These facets typically exhibit different electronic properties compared to lower-index facets like (110) and (111), and their presence could boost alternative reaction pathways.

Again, with the aim of exploring the correlation between surface crystal features and catalytic activity, a recent work by Luc et al. presents the synthesis and catalytic performance of two-dimensional copper nanosheets (CuNSs) designed for the electrochemical reduction of CO to acetate.^92^ These triangular-shaped nanosheets (∼5 nm thick) selectively expose Cu(111) facets and were synthesized through chemical reduction using l-ascorbic acid, CTAB, and HMTA. Stability tests confirmed the robustness of CuNSs and preservation of their metallic character throughout catalysis. Crucially, the study employed OH^–^ adsorption voltammetry to verify the preservation of (111) facets in situ.

Finally, in a recent interesting study by Roldan Cuenya’s group, the role of pulsed current modification on different single-crystal and polycrystalline Cu electrodes was investigated.^87^ They noted a remarkable enhancement in selectivity for ethanol in CO_2_RR at −1.0 V under pulsed electrolysis conditions (which had been explored in previous works by Le Duff et al. in 2017 ^88^ and Kimura et al. in 2018 ^89^), where Cu^+^ was continuously regenerated in situ by careful selection of the anodic potential. The authors proposed that this catalytic enhancement is associated with the presence of specific structural motifs and a Cu_2_O surface layer, which remains stable during the CO_2_ reduction pulses, as evidenced by quasi-in situ X-ray photoelectron spectroscopy (XPS) measurements. Their results indicate that a combination of (100) domains, defect sites, and surface Cu_2_O provides the optimal configuration for enhancing the CO_2_ reduction reaction pathway leading to C_2+_ products.

Table 1 shows a selection of the results of CV experiments under alkaline conditions carried out to probe the crystal facets exposed to the solution of different types of Cu and nanostructured Cu-based electrodes. Although different experimental conditions were used, such as different concentrations of hydroxide salt (0.1 and 1 M) and types of cations (Na^+^, K^+^), no significant variation of the signal in terms of peak potentials was observed. This suggests that, for this type of measurement, the effect of the cation is negligible.

**Table 1:** Summary of Voltammetric Peak Assignment to Cu-Based Crystal Materials and Nanomaterials in Recent Literature

nanomaterial	electrolyte	Cu facets (*hkl*)	*E* vs RHE (V) adsorption	Cu terrace (*hkl*)	*E* vs RHE (V) adsorption	ref
Cu single crystals	0.1 M NaOH	–	–	(110)	–	^47^
		–	–	(100)	–0.15	
		–	–	(111)	+0.12/–0.06	
		–	–	(322)	+0.02	
		–	–	(911)	–0.15	
CuNWs	1 M KOH	(211)	+0.32	–	–	^44^
		(100)	+0.36	–	–	
		(110)	+0.42	–	–	
		(111)	+0.46	(111)	+0.1	
nanostructured Cu film	1 M NaOH	(100)	+0.33	–	–	^90^
		(110)	+0.36	–	–	
		(111)	+0.43	–	–	
nanostructured Cu	0.1 M NaOH	(100)	+0.36	–	–	^84^
		(110)	+0.39	–	–	
		(111)	+0.41	–	–	
Cu flowers	1 M KOH	(100)	+0.34	–	–	^82^
		(110)	+0.37	–	–	
		(111)	+0.42	(111)	+0.1	
CuNWs	0.1 M KOH	(high-index)	+0.316	–	–	^72^
		(100)	+0.362	–	–	
		(110)	+0.395	–	–	
nanostructured stepped Cu	1 M KOH	(100)	+0.34	–	–0.1	^81^
		(110)	+0.38	–	–	
		(111)	+0.44	–	+0.1	
nanostructured Cu oxide	1 M KOH	(100)	+0.37	–	–	^91^
		(110)	+0.41	–	–	
		(111)	+0.48	–	–	
Cu nanocubes	1 M KOH	(100)	+0.35	–	–	^83^
		(110)	+0.38	–	–	
		(111)	+0.45	–	–	
Cu nanosheets	1 M KOH	(100)	+0.36	–	–	^92^
		(110)	+0.40	–	–	
		(111)	+0.47	–	–	
CuNWs	1 M NaOH	(100)	+0.37	(100)	–0.1	^73^
		(110)	+0.41	–	–	
nanostructured Cu	1 M KOH	–	–	–	–	^93^
		(110)	+0.41	–	–	
		(111)	+0.46	–	–	
nanostructured AgCu	0.1 M NaOH	–	+1.05	–	–	^94^

### Underpotential Deposition of Pb on Cu

An alternative voltammetric approach that provides both qualitative and quantitative insight into the electroactive surface is underpotential deposition (UPD). UPD, commonly but not exclusively using Pb^2+^ as a probing metal, provides a useful method not only to account for the different crystal surfaces exposed but also to estimate the electrochemical surface area (ECSA).^48,95,96^ This measurement is crucial for normalizing catalytic activities and comparing catalysts fairly.

Pb UPD on Cu is an electrochemical process in which a monolayer of Pb atoms forms on the Cu surface at potentials more positive then the reversible Nernst potential (*E*°) for bulk Pb deposition.^95,97,98^ This phenomenon occurs because of the strong interaction between Pb and Cu atoms, which promotes the absorption of Pb atoms onto the Cu surface before bulk deposition can occur. This process is sensitive to the surface structure of the catalyst, providing direct insight into the number and nature of active sites. UPD is inherently surface-specific, occurring prior to the nucleation and growth of bulk Pb, and is characterized by its ability to reduce the energy barrier for the initial Pb atom attachment. This enables the formation of a well-ordered, single-atom-thick layer. In contrast to bulk deposition, which results in the formation of multiple layers (Figure 9), UPD allows for precise control of surface coverage, making it a powerful tool for probing and engineering electrochemical interfaces at the atomic level.

**Figure 9:**
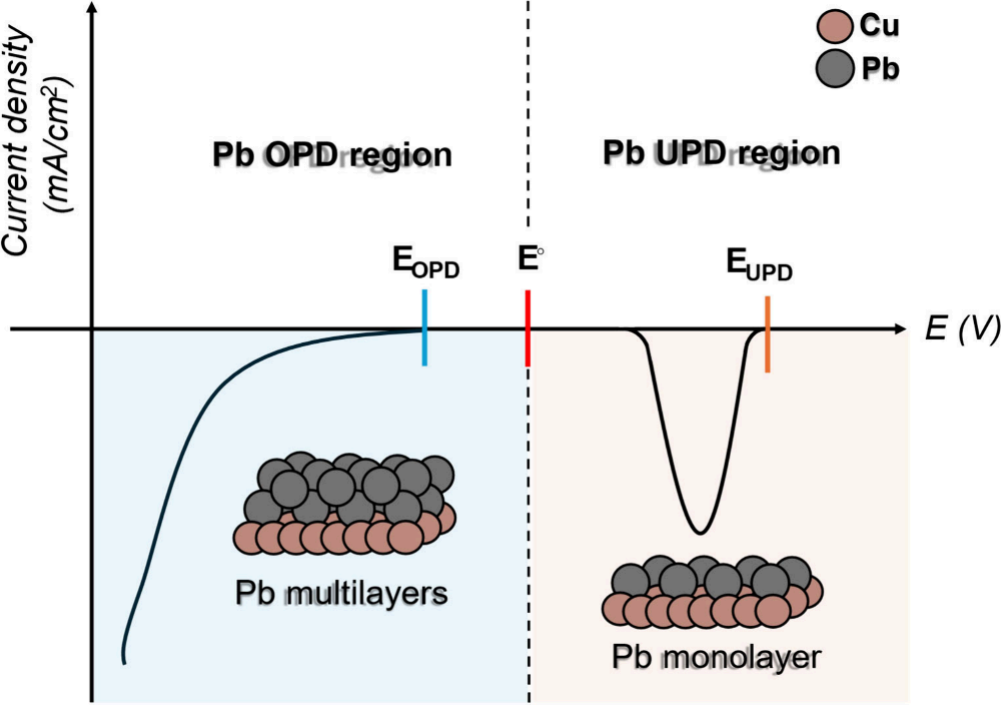
Illustration of overpotential deposition (OPD) (left) and underpotential deposition (UPD) (right). *E*° represents the standard Nernst potential of the deposition process, and *E*_OPD_ and *E*_UPD_ represent the actual values of the potential at which OPD and UPD occur.

In the reverse (oxidative) CV scan, the signal corresponding to the stripping of the Pb monolayer is observed. Quantification of the charge associated with the desorption process allows the estimation of the ECSA.^69^ The process of UPD of Pb on Cu in the presence of Cl ions is very similar on both the (111) and (100) planes. In both cases, Pb UPD displaces the ionic layer and forms a monolayer of fully discharged atoms with essentially the same density as the corresponding fcc plane in bulk Pb. The deposition appears to occur via a single process, the nucleation and growth of Pb islands, but other growth modes cannot be excluded. The presence of Cl ions in the electrolyte improves the kinetics of Pb deposition on both surfaces in the underpotential and overpotential regimes and leads to a negative shift of the UPD average potential by about 0.1 V compared to the (nominal) Cl-free perchloric acid. This shift can be explained by a simple thermodynamic model of the overall Cu/Pb/Cl system. The only real difference in Pb UPD on the two different Cu crystal faces was the absence of an ordered structure for the compact layer on Cu(100) versus the ordered structure observed on Cu(111). This difference appears to be related to a lower mobility of the Pb adatoms due to the greater atomic corrugation of the (100) surface.

Two seminal studies published in 1995 and 1997 by Brisard and Zenati^99,100^ laid the groundwork for much of the recent progress in understanding Pb UPD on copper single-crystal surfaces. These works demonstrated the significant influence of chloride ions on the shape and position of Pb UPD voltammetric features and established that the electrochemical response is fundamentally similar on both UHV-prepared and electropolished Cu surfaces; although slightly broader peaks are observed in the latter, the peak potentials remain effectively unchanged. Building upon this foundation, Sebastián-Pascual and Escudero-Escribano^69^ employed these well-defined CV signatures to deconvolute the distribution of surface facets on Cu electrodes. They proposed that Pb UPD can be employed as a reliable surface characterization tool for tracking structural changes induced by various electrochemical treatments, including pulsed and non-pulsed HER protocols.^69^

Hochfilzer et al. focused on advancing the understanding of surface atomic structures in Cu-based electrocatalysts used for CO_2_ and CO reduction under the condition causing surface restructuring.^101^ The authors used Pb UPD to investigate and map the surface facets of Cu. Figure 10A–C shows UPD of Pb on Cu(111), Cu(100), and Cu(110), respectively, while Figure 10D exhibits the superimposition of the three patterns. It is noteworthy that different Cu facets were shown to have distinct potential windows where Pb UPD occurs, allowing for the in situ examination of polycrystalline Cu electrodes’ predominant facets.^101^ The study also revealed that Pb and Cu form an irreversible surface alloy during UPD, limiting the method’s application to postreaction analysis of surface structures. This work contributed significantly to understanding the dynamic changes in catalyst surfaces during electrochemical reactions and highlighted the potential of UPD as a technique for probing and understanding catalyst surfaces.

**Figure 10:**
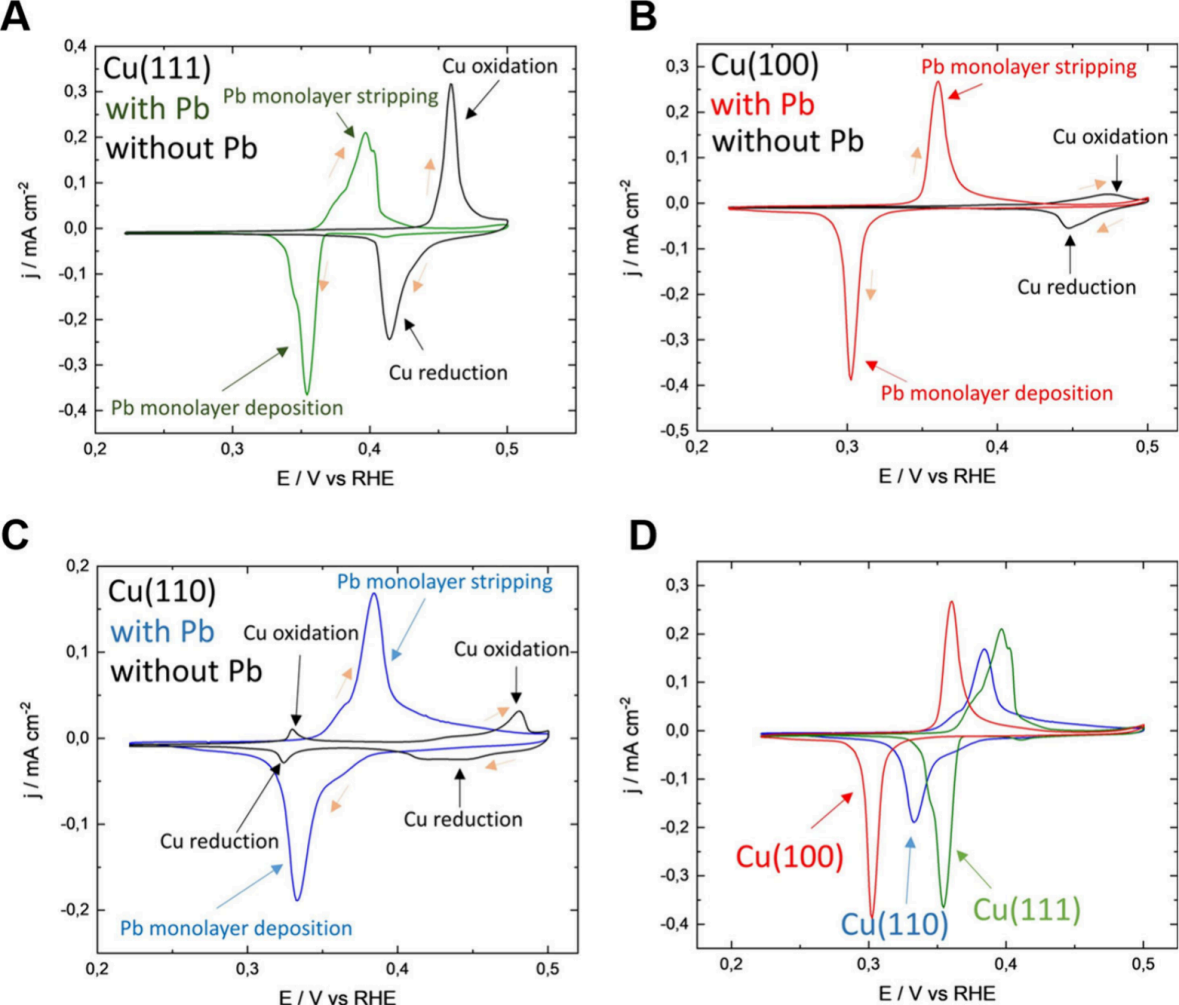
Cyclic voltammograms in 0.1 M KOH and 0.1 M KOH + 1 mM Pb­(NO_3_)_2_ at 10 mV s^–1^ for (A) Cu(111), (B) Cu(100), and (C) Cu(110). (D) Overlaid Pb UPD responses of Cu(111), Cu(100), and Cu(110). Orange arrows indicate the scan direction. Reproduced from ref 101. CC BY-NC-ND 4.0.

A similar approach was used by Couce et al.,^102^ aimed at monitoring and controlling the growth and number of new facets on differently treated Cu. The top panels in Figure 11A–C show the Pb UPD curves on the NaCl-treated Cu surfaces at three distinct oxidative potentials (+1.0, +1.3, and +1.6 V vs SCE) superimposed with the Pb UPD of the original Cu­(poly) (dashed line). An evolution in the CV is observed: the (111) peak at approximately −0.30 V progressively decreases while the peak at −0.35 V vs. SCE, assigned to (310), shows an increase. The bottom panels of Figure 11A–C illustrate the peak deconvolution of Cu­(poly) reconstructed in NaCl at the three distinct oxidation potentials (*E*_ox_). The area fraction of the (111) peak (red area) decreases from 53% in Cu­(poly) (Figure 11B) to 33% at *E*_ox_ = 1.0 V, 45% at *E*_ox_ = 1.3 V, and 33% at *E*_ox_ = 1.6 V. Conversely, the area fraction of the (310) peak (orange area) increases from 19% in Cu­(poly) to 27% at *E*_ox_ = 1.0 V, 34% at *E*_ox_ = 1.3 V, and 45% at *E*_ox_ = 1.6 V. This result suggests that Cl mainly reduces the large (111) terrace domain areas in Cu­(poly) and promotes the formation of (310) or *n*(100) × (110) domains.

**Figure 11:**
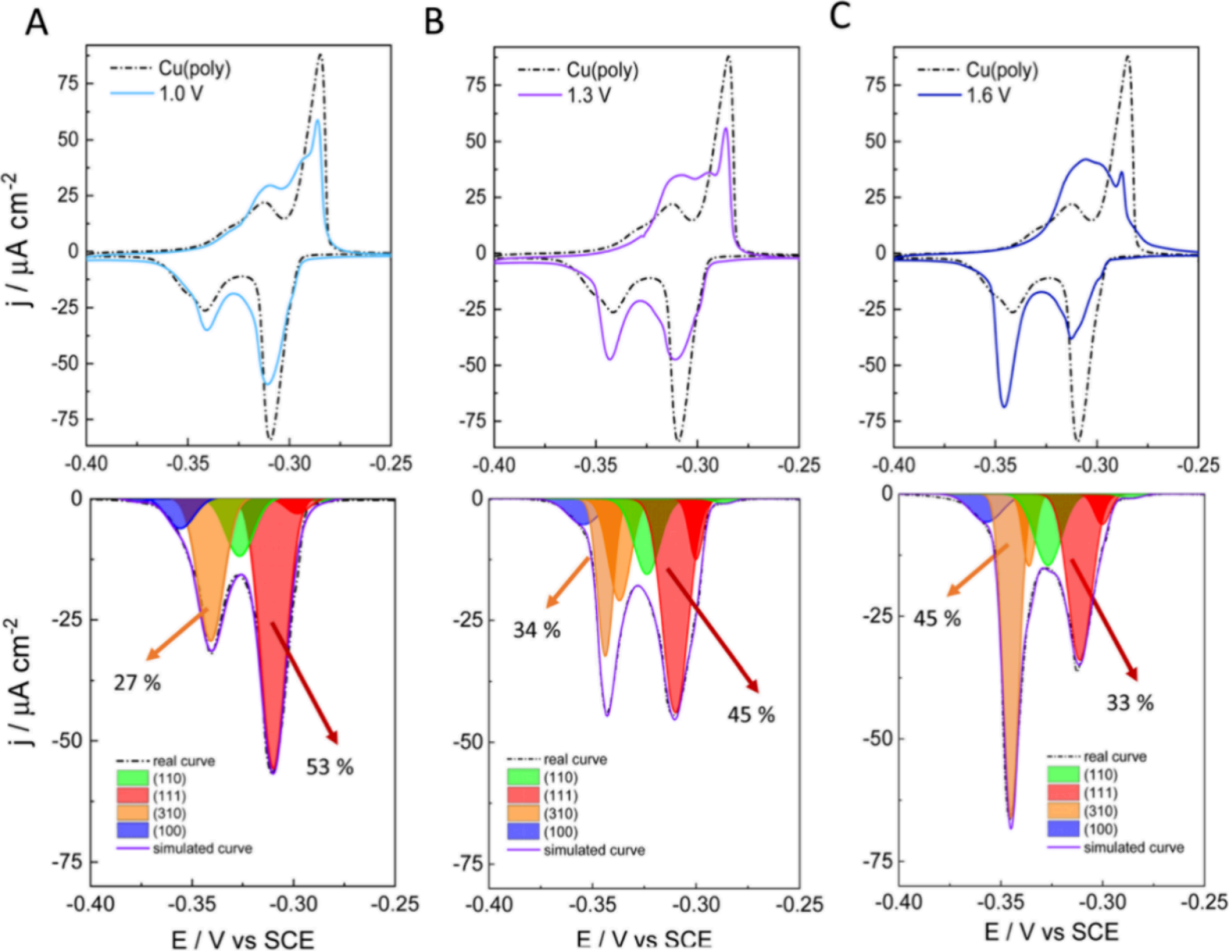
Pb UPD CVs on restructured Cu surfaces in 2 mM Pb­(ClO_4_)_2_·(H_2_O), 2 mM NaCl, 1 mM HClO_4_, and 0.1 M KClO_4_ at 5 mV s^–1^. The Cu surfaces were restructured in a solution of 0.1 M NaCl by performing three cycles at 500 mV s^–1^ between −1 V and (A) 1.0 V, (B) 1.3 V, and (C) 1.6 V vs SCE. The top panels show the Pb UPD CVs of the restructured Cu surfaces. The bottom panels show the peak deconvolutions of the cathodic curves. The arrows indicate the percentage of integrated areas corresponding to (310) or (111) facets. Reproduced from ref 102. CC BY-NC 3.0.

In 2021 Plaza-Mayoral et al. reported the preparation of high-surface-area bimetallic nanostructures made from Cu and Au using an electrodeposition method in a deep eutectic solvent (DES).^103^ The researchers utilized a co-electrodeposition technique in a DES composed of choline chloride and urea to create nanostructured Cu–Au materials. This method allowed for control over the size, morphology, and composition of the nanostructures. The UPD analysis indicated a significant increase in surface area compared to that of flat polycrystalline Cu or Au, which enhances their catalytic efficiency. The use of DES for the synthesis of these materials is highlighted as an environmentally friendly alternative to traditional methods, reducing the need for toxic chemicals and high-energy processes. UPD was also used in another work by Plaza-Mayoral et al.^104^ to measure the ECSA of Cu–Ag nanostructured electrocatalysts for CO_2_ reduction (Figure 12). The study differentiates the effects of physical surface area from the catalytic material’s intrinsic properties. This is particularly important when comparing catalysts with different compositions or morphologies. For instance, the study found that despite similar ECSA values, catalysts with different Cu/Ag ratios exhibit different catalytic behaviors and product selectivities. Although the addition of Ag only slightly modifies the ECSA, it significantly affects the CO_2_RR product distribution by suppressing HER and favoring the formation of alcohols and oxygenates.^104^ The UPD data provide evidence that the surface structure and available active sites are directly influenced by the incorporation of Ag into the Cu nanostructures.

**Figure 12:**
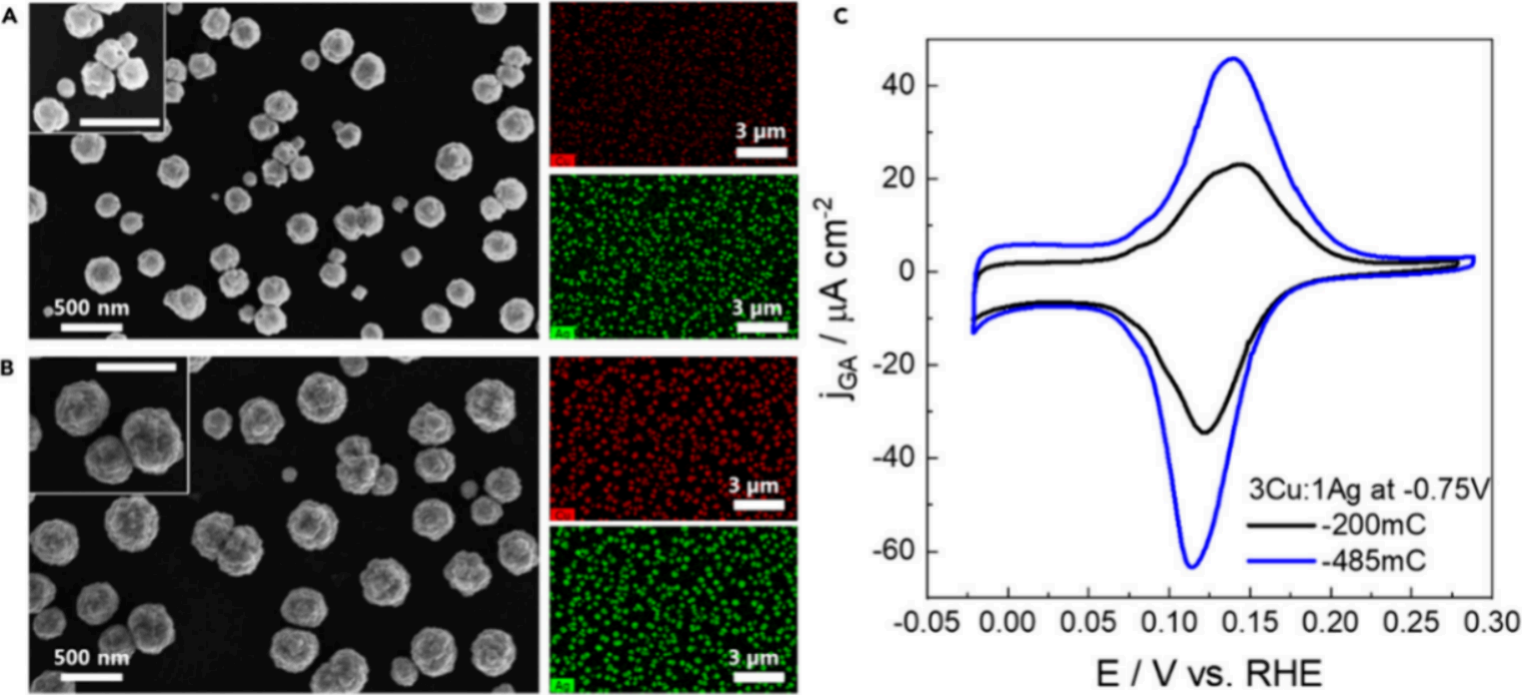
(A, B) SEM images of Cu/Ag-based catalysts obtained under different deposition conditions, with the corresponding EDS maps: (A) 200 mC; (B) 485 mC. (C) Pb UPD of the nanostructures in (A) and (B) using 2 mM Pb­(ClO_4_)_2_·(H_2_O), 2 mM NaCl, 1 mM HClO_4_, and 0.1 M KClO_4_ at 5 mV s^–1^. Reproduced with permission from ref 104. CC BY-NC 4.0.

A very recent work by Pascual-Llorens et al. that appeared in 2025 analyzed the modification of single-crystal Cu(111) surfaces with the aim to achieve controlled formation of nanostructures on Cu(111) using Cl ions and a square-wave potential method.^105^ The application of potential pulses between −1.3 and 0.5 V vs SCE at a frequency of 1 Hz initially (45–150 s) led to the formation of hexagonal micro- and nanoclusters uniformly distributed on the Cu(111) surface that then transitioned from hexagonal to triangular pyramid shapes over time. Extended potential pulse applications (300–3600 s) led to larger, more defined hexagonal and later triangular pyramidal structures, indicating that structure and crystal growth are both time- and morphology-dependent. SEM and CV analysis revealed that chloride ions significantly influence the structure and orientation of crystalline growth, favoring the formation of high-index facets such as (210) and (310). The controlled electrochemical modification process allowed for systematic tuning of the Cu surface, showing that the growth and morphology of Cu crystalline structures can be finely tuned through the manipulation of electrochemical conditions and substrate orientation. Figure 13A provides a detailed analysis of the changes observed in the Cu(111) surface when modified for 300 s using the square-wave potential method in a 0.1 M NaCl solution. The Pb UPD pattern highlights three signals: P1 referring to the (111) facet, P2 due to (110) and defects, and P3 assigned to the (310) and (*n*10) facets. The SEM images in Figure 13B,C reveal large, well-defined hexagonal microstructures with sharp edges, which suggest that the surface has undergone significant reconfiguration under the influence of chloride ions and the applied potential. These microstructures are surrounded by smaller, less defined structures, illustrating the progressive nature of the Cu redeposition process during the electrochemical modification. Figure 13D provides theoretical simulations of particles with different facet ratios, helping to interpret the SEM observations.

**Figure 13:**
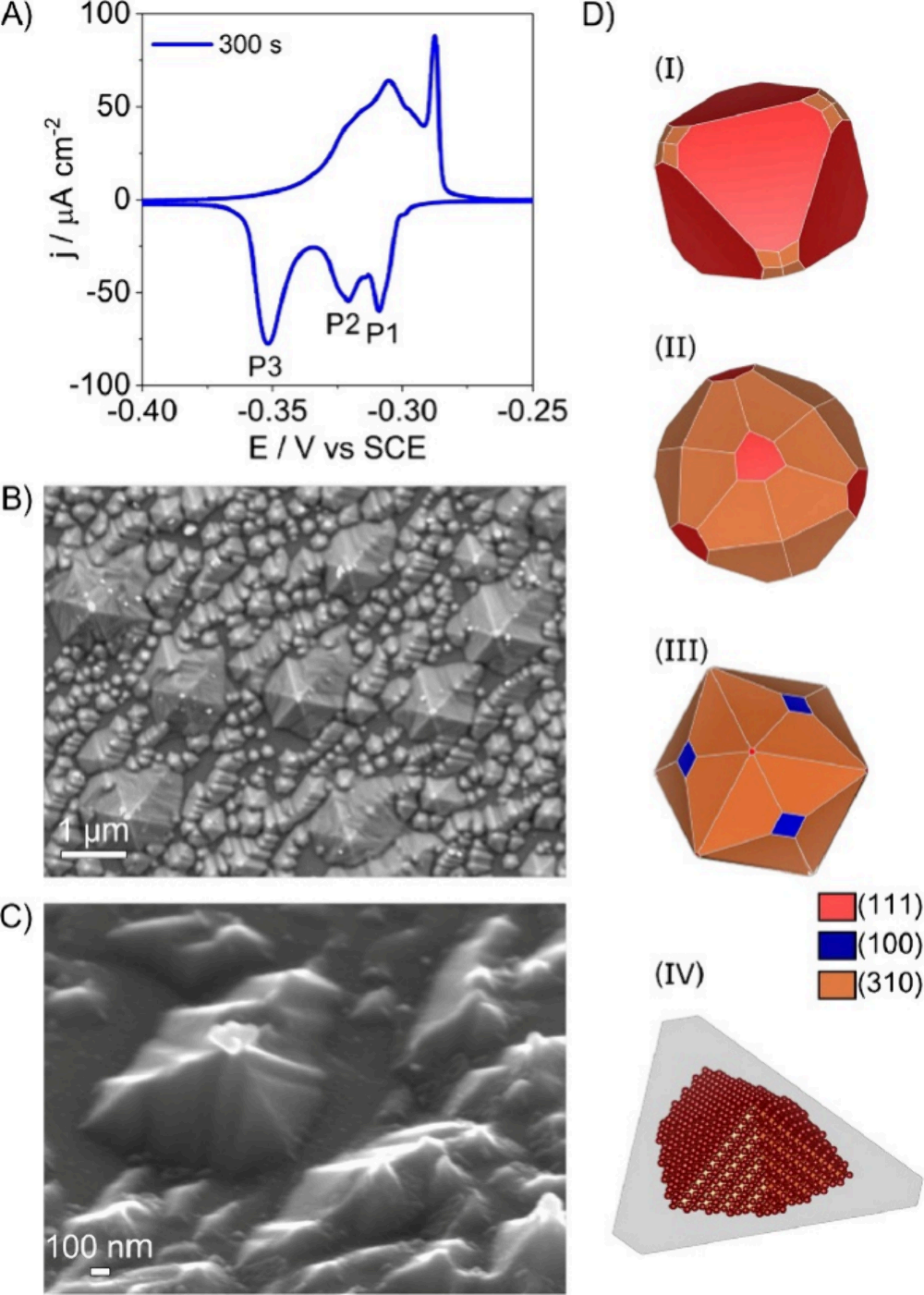
(A) Pb UPD CV of a Cu(111) surface that was modified for 300 s, recorded at 5 mV s^–1^, The electrolyte solution consisted of 0.1 M KClO_4_ + 2 mM NaCl + 2 mM Pb­(ClO_4_)_2_·H_2_O + 1 mM HClO_4_. (B, C) SEM images of the Cu(111) surface modified for 300 s. (C) was recorded at an angle of 45° between the electron beam and the surface normal. (D) Simulated particle morphologies with varied (111), (100), and (310) facets. Reproduced from ref 105. CC BY 4.0.

## Electrochemical Characterizations of Crystalline Ag Electrodes and Ag-Based Nanomaterials

Ag electrodes and related nanostructures are increasingly recognized for their remarkable properties in the field of electrocatalysis.^106–111^ These materials offer a unique combination of high conductivity, chemical stability, and catalytic activity that make them ideal for various electrochemical applications.^112–115^ In alkaline media, the electrochemical behavior of Ag is especially interesting because of its dynamic surface processes.^116,117^ The interaction of Ag with hydroxide ions in alkaline solutions leads to the formation of Ag_2_O species, which can undergo reversible redox transitions. This electrochemical fingerprint is crucial for the investigation of processes such as the oxygen reduction and evolution reactions, where the kinetics and mechanisms are strongly influenced by the surface state of the electrode.^118^ Moreover, Ag nanoparticles enhance these properties due to their increased surface area and the presence of large amounts of active sites.^119^ These nanoparticles can be tailored in size and shape, thus modifying their interaction with the electrolyte and improving their electrocatalytic performance.^36,120–121,122^ Understanding the electrochemical fingerprint of the Ag surface in alkaline media involves studying the surface-adsorbed species and the potential-dependent changes in surface chemistry.^123^ These studies are essential for optimizing the design of Ag-based catalysts and improving their efficiency in energy conversion systems, such as fuel cells and electrolyzers.^124,125^ By focusing on the electrochemical properties of Ag in these environments, researchers can develop more active, selective, and durable electrocatalytic materials. As done for Cu, we present here some significant works that explore the nature of the Ag electrode surface using OH^–^ adsorption and UPD.

In 2004, Horswell et al. showed the features of OH^–^ adsorption on Ag crystallographic surfaces (111), (110), and (100).^126^ The voltammetries showed distinct peaks related to OH^–^ electrosorption followed by submonolayer oxide formation, with each Ag plane exhibiting a unique electrochemical signature, as shown in Figure 14A, which displays the fingerprint of a Ag single crystal. Ag(110) showed OH^–^ electrosorption at around −0.9 V, Ag(100) at −0.7 V, and A (111) at 0.6 V vs Hg/HgO (0.1 M NaOH). Interestingly, in Figure 14B the same CVs are relatively shifted on the potential scale and displayed against their potential of zero charge (*E*_pzc_) value, resulting in overlap of peaks. The differences in peak positions are attributed to the variations in *E*_pzc_ across the three crystal facets of Ag. This outcome can be rationalized by considering that *E*_pzc_ at a metal–electrolyte interface is affected by the metal work function (Φ) and the changes in surface potentials of both the metal (δM) and the solvent (δS) upon their interaction, as defined by eq 1:

$$E_{\mathrm{pzc}}=\Phi +\mathrm{\delta M}-\mathrm{\delta S}$$ 1

A comprehensive analysis of published data by Trasatti demonstrates a linear relationship between *E*_pzc_ and the work function of Ag single-crystal planes with a slope close to 1.^127,128^ This indicates that despite variations in the interfacial parameters δM and δS across different planes, the shift in *E*_pzc_ is primarily governed by the work function. Thus, the alignment of peak positions on the *E*_pzc_ potential scale suggests that the initiation of OH^–^ electrosorption on Ag surfaces is directly linked to the work functions of these surfaces. Further analyses such as low-energy electron diffraction (LEED) and reflection high-energy electron diffraction (RHEED) provided evidence of different structural changes on the Ag surfaces. Ag(110) displayed ordered surface structures, whereas Ag(111) and Ag(100) exhibited more disordered or nonuniform layers. Moreover, second harmonic generation (SHG) studies highlighted differences in surface symmetry and adsorption-induced changes, with clear variations between the different crystal faces. When the potential is increased beyond the anodic peak labeled B in the CV profile, the current sharply rises for all Ag planes. If the positive limit of the CV exceeds the Ag/Ag_2_O equilibrium potential (*E*_eq_) of 0.18 – 0.059 log­[OH^–^] V vs Hg/HgO/0.1 M NaOH (at 25 °C), the CVs become irreversible, as shown in Figure 14, indicating the formation of bulk Ag_2_O. It is important to note that unlike the OH^–^ electrosorption process, which is influenced by the potential difference (*E* – *E*_pzc_) and therefore varies with different Ag planes, the formation of Ag_2_O is governed by the potential difference (*E* – *E*_eq_) and occurs at roughly the same potential for different Ag crystal planes. The study not only elucidates the specifics of hydroxide interaction with Ag surfaces but also highlights the importance of surface orientation in modifying the electrochemical reactivity and structural properties. Already in 1997 Hoshi et al. investigated how the surface orientation of Ag single crystals affects the electrochemical reduction of CO_2_.^116^ Using macroelectrolysis in a 0.1 M KHCO_3_ solution, the authors analyzed the formation rates of products such as CO, HCOO^–^, and H_2_ across different crystallographic planes of Ag. The importance of this paper relies also on the characterization of single-crystal surfaces through adsorption of Cl^–^ and fingerprints in bicarbonate baths. Indeed, voltammograms of Ag(111), Ag(110), and Ag(100) in 0.01 M NaCl were reported. These blank voltammetries showed two different groups of peaks; the broad peaks were assigned to the adsorption of a random adlayer of Cl^–^, and conversely, the sharp peaks at more positive potentials were attributed to the formation of ordered AgCl adlayers. The authors characterized the single-crystal electrodes in 0.1 M KHCO_3_ electrolyte, showing similar trends in terms of pzc observed in more recent work just reported in this review.

**Figure 14:**
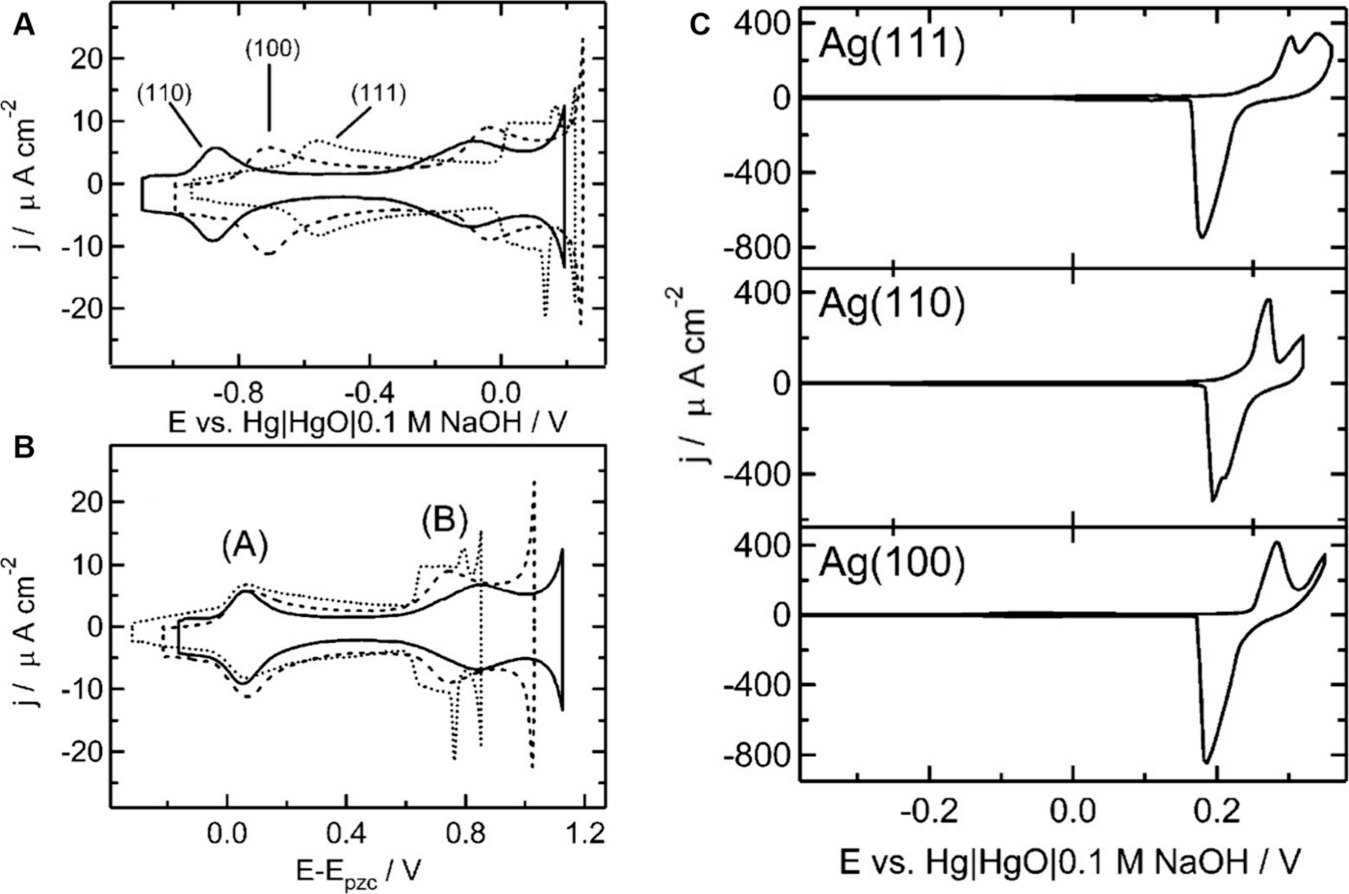
(A, B) CVs of Ag(111) (dotted line), Ag(110) (solid line), and Ag(100) (dashed line) in 0.09 M NaF + 0.01 M NaOH with potential scale referred to (A) Hg/HgO/0.1 M NaOH and (B) the respective *E*_pzc_. (C) CVs of Ag(111), Ag(110), and Ag(100) in 0.09 M NaF + 0.01 M NaOH with an extended anodic potential limit. Reproduced from ref 126. Copyright 2004 American Chemical Society.

Similar CV patterns were obtained by Jovic et al., as shown in Figure 15, where the effects of NaOH concentration (A) and surface structure (C) are exemplified.^129^ Figure 15A shows the effect of concentration of OH^–^: each voltammogram displays two asymmetric pairs of anodic and cathodic peaks, occurring between −0.7 and −0.5 V and between −0.1 and 0.1 V, respectively. The initial anodic peak at the more negative potential range is associated with the adsorption of OH^–^ ions, characterized by a sharp peak followed by a broad shoulder. This pattern suggests a complex adsorption process, possibly indicating a phase transformation at this point.^130,131^ The observed peak potential difference of approximately 60 mV suggests a single electron transfer in the electrochemical reaction, affirming complete charge transfer between the OH^–^ ions and the Ag surface. The second set of peaks, characterized by a combination of overlapping broad and sharp features, indicates another phase transformation, likely resulting in the formation of a AgOH monolayer, as suggested by other researchers.^132^

**Figure 15:**
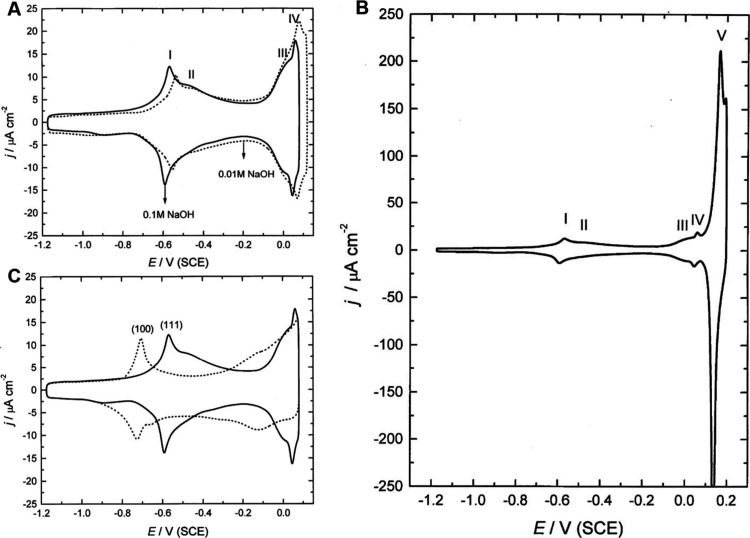
(A) CVs recorded on Ag(111) in solutions of different concentrations of NaOH at a sweep rate of 50 mV s^–1^. (B) CV recorded on Ag(111) in a solution of 0.1 M NaOH at a sweep rate of 50 mV s^–1^. (C) CVs recorded on Ag(111) and Ag(100) in a solution of 0.1 M NaOH at a sweep rate of 50 mV s^–1^. Reproduced with permission from ref 129. Copyright 1999 Elsevier.

Figure 15B illustrates that after the second adsorption peak at 0.06 V, a distinct set of anodic/cathodic peaks becomes evident, with the oxidation peak reaching approximately 180 mC/cm^2^. This suggests the oxidation of slightly less than one monolayer of silver. When the electrode is cycled up to 0.2 V vs SCE within this region, there are no changes in the “double layer” region of the voltammogram, indicating reversible processes. Figure 15C compares the CVs of the (111) and (100) faces of Ag in 0.1 M NaOH at the same sweep rate. The adsorption of OH^–^ ions occurs at more negative potentials on the (100) face, which favors anion adsorption more. While the initial adsorption peaks appear similar for both crystal faces, the broad shoulder is less pronounced on the (100) surface. Near 0.0 V, the voltammetric response on the (100) face differs from that on the (111) face; notably, the sharp peak indicating a phase transformation on the (111) face is absent on the (100) face. Nevertheless, the total charge in the anodic portion of the voltammogram, adjusted for the double-layer charge, aligns well with the charge needed to form a Ag–OH monolayer on the Ag(100) face.

With regard to Ag nanostructures, Bansal et al. showed how their electrochemistry differs with respect to bulk Ag and is affected by the nanocrystal geometry and structure.^133^ They reported the CV of a large-scale Ag electrode in a 1 M NaOH solution, demonstrating typical Ag behavior. During the positive sweep, a pronounced double-layer charging occurs between 1.0 and 0.0 V vs Ag/AgCl. A prominent oxidation peak emerges around 0.34 V, with a preceding smaller peak at approximately 0.25 V, likely related to the transition between Ag^0^ and its oxidized form Ag^+^, potentially forming either hydroxide or Ag_2_O compounds. The exact composition of the oxide layer remains uncertain, possibly involving both hydroxides and Ag_2_O. The return sweep shows a distinct cathodic peak indicative of the reverse transition. In addition, they collected the CV behavior for Ag nanostructures, such as nanocubes, nanospheres, and nanoprisms. Notably, the oxidation onset for Ag nanospheres begins at a less positive potential compared with cubes and prisms. On the reverse sweep, there is a variation in the hysteresis/overpotential of the reduction peak across different samples, with the reduction peaks shifting to more negative potentials in the order of nanocubes, nanospheres, and then nanoprisms. This suggests that the oxides formed on Ag nanoprisms are the most stable and challenging to reduce.^133^

Kuang et al. explored the enhancement of CO selectivity in CO_2_ electroreduction using Ag microtubular gas-diffusion electrodes modified by surface reconstruction.^134^ The authors focused on improving the performance of the CO_2_ reduction reaction by creating a porous microparticle Ag-based electrode using an in situ electrochemical oxidation–reduction method. The Ag hollow fiber gas-diffusion electrode (HFGDE) was engineered with a porous structure to enhance mass transport and triple-phase-boundary interactions, crucial for effective CO_2_RR. The unique fabrication involved an electrochemical method to form a micro/nanostructured surface, enhancing the catalytic properties. The electrode exhibited a Faradaic efficiency for CO, significantly outperforming traditional flat-surface electrodes. The surface characterization of the HFGDE was performed through CV in alkaline media, and XRD was used to assess the presence of Ag_2_O.

### UPD of Pb and Cu on Ag

UPD of Pb on Ag is analogous to the process described for Cu electrodes, with the difference that it occurs at different potentials. As previously mentioned, this approach provides valuable information about crystal facet distribution and ECSA involving the deposition of a submonolayer of Pb onto Ag surfaces slightly below the Pb reduction potential, followed by scanning to positive potentials to dissolve the Pb and measure the oxidative current.

Vitanov et al. performed a pioneering study of Pb UPD on electrolytically grown Ag(111) and Ag(100) single-crystal surfaces, with particular focus on the influence of step density and structural transformations in the Pb adsorbate layer.^135^ On Ag(111) surfaces, the behavior of Pb UPD is strongly dependent on the density of atomic steps. At low step densities, Pb deposition exhibits slow structural transformations, with two prominent peaks in voltammetry, indicating relatively stable submonolayer structures. As the step density increases, these transformations become faster, and new peaks emerge in the CV, signifying that the Pb adlayer undergoes more dynamic rearrangements. The transformation is interpreted as a partial exchange between Pb atoms and surface Ag atoms, occurring preferentially at step sites that act as active centers for reorganization. The higher the step density, the faster is the formation of compact, stable Pb adlayers and the more pronounced are the associated voltammetric features (e.g., new adsorption/desorption peaks). In contrast, Ag(100) surfaces show different UPD behavior. No significant structural transformations were observed, regardless of step density. CV curves showed only minor kinetic effects, such as peak splitting or changes in intensity, suggesting that Pb adsorption occurs more rigidly without reordering. The lack of transformation is attributed to either thermodynamic instability of Pb–Ag surface alloys on (100) or to kinetic limitations such as reduced Pb atom mobility or stronger competing anion adsorption (e.g., ClO_4_^–^). In summary, UPD of Pb on Ag(111) is characterized by step-density-dependent structural transformations, while on Ag(100) the Pb adlayer forms more rigidly, without such rearrangements. This highlights the importance of the crystallographic orientation and surface defects in governing UPD dynamics.

Van Cleve et al. studied spherical and cubic Ag nanoparticles with a diameter of around 40 nm supported on carbon.^136^ These Ag nanoparticles were synthesized and tested for their ORR activity under alkaline conditions. Despite initial hypotheses suggesting that cubic nanoparticles might exhibit higher reactivity due to a higher proportion of Ag(100) facets (which bind oxygenated intermediates more strongly), it was found that spherical nanoparticles slightly outperformed the cubic ones. The difference in activity between the shapes was linked to the abundance of surface facets. Spheres, which presented a mix of Ag(111) and Ag(100) facets, showed better performance compared with cubes primarily exhibiting Ag(100) facets. This contradicted the expectation that stronger binding of intermediates on Ag(100) would enhance the ORR activity. Spherical nanoparticles showed a significant presence of Ag(111) facets, which bind intermediates less strongly and thus remain more catalytically active under the ORR conditions. In contrast, cubic nanoparticles, with a higher proportion of Ag(100) facets, likely experienced site poisoning at high potential due to overly strong binding of intermediates. The slightly superior activity of the Ag spheres was consistent with their mixed facet composition, which provided a more balanced interaction with ORR intermediates. This work effectively utilized UPD to measure the ECSA and the impact of surface facet distribution on the catalytic performance of Ag nanoparticles in the ORR, contributing valuable insights into the design of more effective Ag-based catalysts for alkaline fuel cells.

Thiel et al. explored the UPD of Cu on various Ag substrates using CV, potential step experiments, and XPS,^137^ showing how modifications of the electronic properties of Ag due to different underlying substrates like Au(111) and the presence of organic molecules like thymine affect Cu deposition. Cu UPD successfully occurs on Ag monolayers deposited on Au(111), forming a Au–Ag–Cu sandwich structure in the absence of specific anions and thymine. This UPD behavior on Ag differs significantly from its occurrence on Au(111), where it typically requires the presence of specifically adsorbed anions or thymine. The UPD process is highly sensitive to the electrolyte composition. It proceeds in the presence of perchlorate ions but not sulfate ions, highlighting the influence of anion adsorption. No UPD is observed on bulk Ag or a second Ag monolayer, which has electronic properties similar to those of bulk Ag. Thymine affects UPD dramatically: it promotes UPD on Au(111) but inhibits it on silver. This is likely due to the differences in thymine’s adsorption states on these metals at the potential used for Cu deposition. The results underline the complexity of UPD, which depends on a delicate balance of the substrate properties, electrolyte composition, and molecular adsorption. The presence or absence of thymine, alongside the type of anions in the electrolyte, plays a critical role in determining the feasibility and nature of UPD on different substrates. This study extends the understanding of metal deposition in electrochemistry, particularly the nuanced interactions that determine UPD behavior on different substrates in varying chemical environments.

Finally, it is useful to consider the study by Liu et al. that explored the electrocatalytic reduction of CO_2_ to CO using triangular Ag nanoplates (Tri-Ag-NPs), shown in Figure 16A.^138^ Figure 16B–D shows Pb OPD and UPD on three different Ag electrodes: Ag nanoplates, Ag nanoparticles, and bulk Ag, respectively. Pb UPD (−0.20 and −0.30 V vs SCE) was used to calculate the ECSA, revealing a higher ECSA for Ag nanoplates. The authors emphasized the shape-dependent catalytic activity, showing that Tri-Ag-NPs exhibit significantly enhanced current density, Faradaic efficiency, and energy efficiency compared to similarly sized Ag nanoparticles and bulk Ag. This superior performance was attributed to the optimal facet and edge-to-corner ratio provided by the triangular shape, which facilitates the electrochemical conversion process at lower overpotentials. Theoretical calculations indicated that the enhanced activity and selectivity of Tri-Ag-NPs are due to their shape-controlled structure, particularly the Ag(100) facet. The study also confirms the durability of Tri-Ag-NPs over 7 days, maintaining high selectivity and activity without degradation. Shape-controlled synthesis offers a promising method to enhance the catalytic performance of metal catalysts.

**Figure 16:**
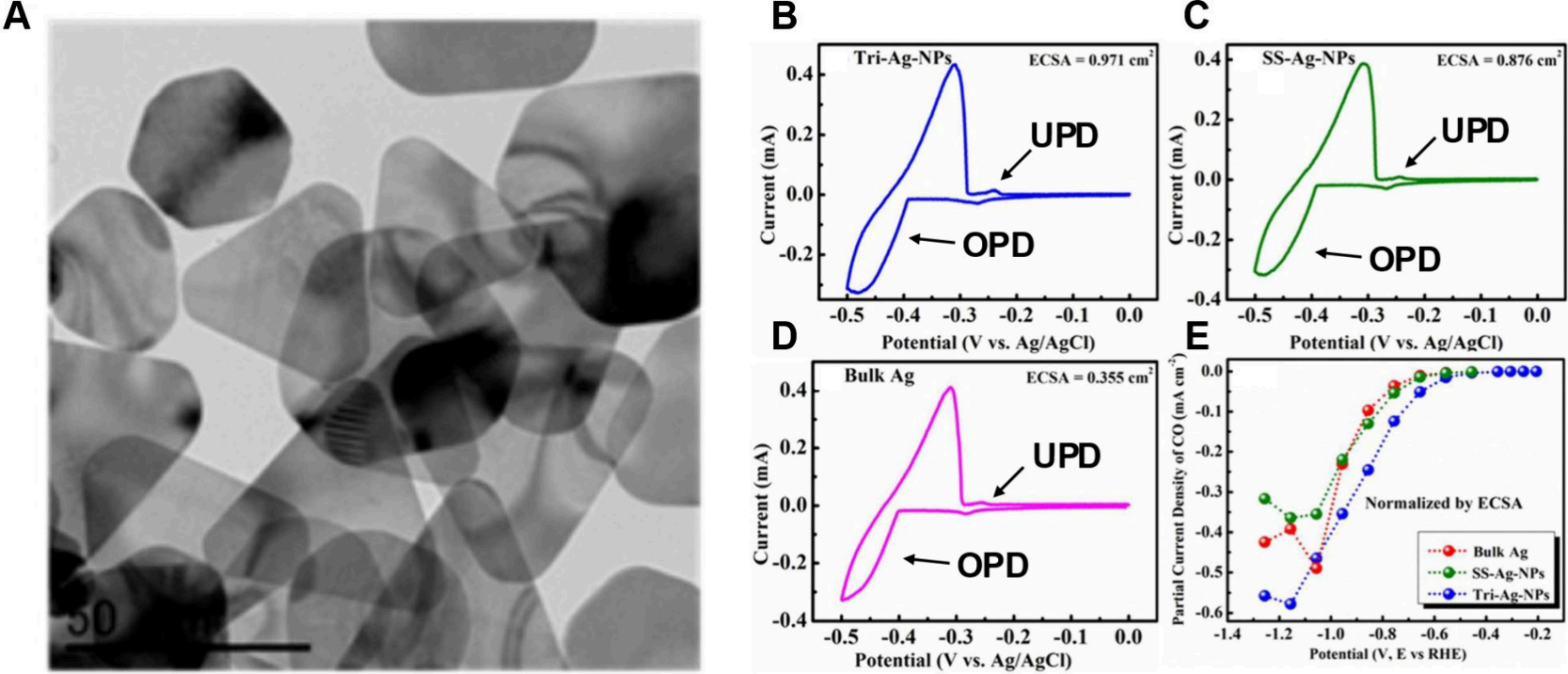
(A) TEM image of Tri-Ag-NPs. (B–D) Cyclic voltammograms of UPD and bulk deposition of Pb in 5 mM Pb­(NO_3_)_2_, 10 mM HNO_3_, and 10 mM KCl solution at 10 mV·s^–1^ for (B) Tri-Ag-NPs, (C) SS-AgNPs, and (D) bulk Ag. (E) CO current density normalized by ECSA. Reproduced from ref 138. Copyright 2017 American Chemical Society.

## Conclusion and Perspective

The voltammetric characterization of metallic interfaces through the OH^–^ electrosorption process and UPD of exogenous metals has become, over the past few years, a fundamental technique for providing comprehensive insights into the surface properties of different metallic electrodes such as surface crystal structures and roughness parameters that are crucial to evaluating their activity as electrocatalysts. In this review, we have emphasized the added value of these cost-effective approaches and reported on recent innovative studies that employed CV to gain a deeper understanding of the composition and structure of Cu and Ag nanomaterials used for electrocatalytic purposes. The data surveyed and collected in Table 1 indicate a good degree of reproducibility of the results across different laboratories worldwide in terms of peak positioning on a potential scale. However, further work is still needed to quantify parameters such as defect density and couple the voltammetric data with other surface-sensitive techniques such as STM. Moreover, fundamental answers on the atomic-scale structural modifications that occur at the metal surface layers during the catalytic process are still missing, thus hindering an in depth understanding of the electrocatalyst’s mechanism of action. In our opinion, advanced computational modeling combined with high-resolution experimental techniques such as single-atom spectroscopy and time-resolved X-ray diffraction should represent the next interdisciplinary approach for improving electrocatalysis’ innovation based on Cu and Ag electrodes. These methods will indeed potentially enable deeper insights into the dynamic processes occurring at the electrochemical interfaces during reactions. Machine learning and artificial intelligence in the design and optimization of these nanostructured electrodes will further accelerate the discovery of optimal configurations for specific catalytic processes. Environmental sustainability together with production scalability are also crucial aspects, and therefore, the development of greener synthetic methods and recycling of precious materials represent mandatory scientific and technological objectives to leverage this material toward real application and social impact. Our perspective promotes sound holistic approaches where detailed understanding of the materials at the nanoscale is coupled with innovative engineering strategies.
